# Distinct lipid bilayer compositions have general and protein-specific effects on K^+^ channel function

**DOI:** 10.1085/jgp.202012731

**Published:** 2021-01-13

**Authors:** Laura-Marie Winterstein, Kerri Kukovetz, Ulf-Peter Hansen, Indra Schroeder, James L. Van Etten, Anna Moroni, Gerhard Thiel, Oliver Rauh

**Affiliations:** 1 Membrane Biophysics, Technische Universität Darmstadt, Darmstadt, Germany; 2 Department of Structural Biology, Christian-Albrechts-Universität, Kiel, Germany; 3 Department of Plant Pathology and Nebraska Center for Virology, University of Nebraska Lincoln, Lincoln, NE; 4 Department of Biosciences and Consiglio Nazionale delle Ricerche, Istituto di Biofisica Milano, Università degli Studi di Milano, Milano, Italy

## Abstract

It has become increasingly apparent that the lipid composition of cell membranes affects the function of transmembrane proteins such as ion channels. Here, we leverage the structural and functional diversity of small viral K^+^ channels to systematically examine the impact of bilayer composition on the pore module of single K^+^ channels. In vitro–synthesized channels were reconstituted into phosphatidylcholine bilayers ± cholesterol or anionic phospholipids (aPLs). Single-channel recordings revealed that a saturating concentration of 30% cholesterol had only minor and protein-specific effects on unitary conductance and gating. This indicates that channels have effective strategies for avoiding structural impacts of hydrophobic mismatches between proteins and the surrounding bilayer. In all seven channels tested, aPLs augmented the unitary conductance, suggesting that this is a general effect of negatively charged phospholipids on channel function. For one channel, we determined an effective half-maximal concentration of 15% phosphatidylserine, a value within the physiological range of aPL concentrations. The different sensitivity of two channel proteins to aPLs could be explained by the presence/absence of cationic amino acids at the interface between the lipid headgroups and the transmembrane domains. aPLs also affected gating in some channels, indicating that conductance and gating are uncoupled phenomena and that the impact of aPLs on gating is protein specific. In two channels, the latter can be explained by the altered orientation of the pore-lining transmembrane helix that prevents flipping of a phenylalanine side chain into the ion permeation pathway for long channel closings. Experiments with asymmetrical bilayers showed that this effect is leaflet specific and most effective in the inner leaflet, in which aPLs are normally present in plasma membranes. The data underscore a general positive effect of aPLs on the conductance of K^+^ channels and a potential interaction of their negative headgroup with cationic amino acids in their vicinity.

## Teaser

Winterstein et al. use the natural diversity of small viral K^+^ channels to address the impact of phospholipid bilayer composition on channel function. Single-channel recordings reveal a stimulating effect of anionic phospholipids on channel conductance and protein-specific effects on gating.

## Introduction

Ion channels are an important class of proteins that are present in every cellular membrane for the regulated and selective transport of ions. In the last two decades, the structure/function correlates of these proteins have been intensively studied. In particular, the class of K^+^ channels, which catalyze the flux of K^+^ in a highly selective manner (e.g., Hille, 2001), has been examined, and many of their functional aspects such as gating and selectivity are now known in great detail down to the atomic level (Doyle et al., 1998; Choe, 2002). Even though K^+^ channels are, like all membrane proteins, in intimate contact with the lipid bilayer, most of the structure/function aspects of these channels have been examined without or with little consideration for their lipid environment. However, the lipid environment can vary between different cells or between the plasma membrane and the membrane of organelles (Coskun and Simons, 2011). On a microscopic level, the lipid composition of the inner leaflet of the plasma membrane differs from the outer one (Fadeel and Xue, 2009), and membranes undergo a dynamic phase separation of lipid components and cholesterol into ordered and liquid-disordered domains (Simons and Ikonen 1997; Honigmann et al., 2010; Lingwood et al., 2008).

An increasing number of reports in the literature indicate that different lipids and different lipid domains as well as the presence/absence of cholesterol affect the activity of K^+^ channels. Examples that illustrate the plethora of lipid effects on K^+^ channel activity include parameters such as bilayer thickness (Yuan et al., 2004; Rusinova et al., 2014) and cholesterol content (Singh et al., 2011). It is also well established that the aforementioned clustering of cholesterol and/or sphingolipids forms so-called lipid rafts (Simons and Ikonen, 1997). These rafts form ordered structures within the membrane that locally alter the membrane’s physical-chemical properties (Brown and London, 2000), leading to an impact on the function of membrane proteins including ion channels (Bukiya et al., 2011; Dopico et al., 2012).

Other components of the bilayer that affect channel function are anionic lipids. Most important is the monoanionic lipid phosphatidylserine (PS) and the polyanionic lipid phosphatidylinositol 4,5-bisphosphate (PIP_2_). In the plasma membrane of healthy cells, both molecules are exclusively located in the inner leaflet (Fadeel and Xue, 2009), where PS contributes up to 30% (Rivel et al., 2019) and PIP_2_ contributes 1% to the total phospholipid (PL) content (Suh and Hille, 2008).

In the last two decades, it has been shown that PIP_2_ binds to distinct structural domains of Kir and Kv channels at the membrane/cytosol interface (Suh and Hille, 2008). This binding stabilizes the open state conformation and regulates in a physiological context cell metabolism, heart pacing, and vascular tone (Shyng et al., 2000; Singh et al., 2011).

The complexity of functional impacts that monoanionic lipids have on K^+^ channels is evident from studies with the model channel KcsA, which show that anionic lipids affect structure and function of this channel in different ways. On a macroscopic level, anionic PLs increase the thermal stability and the folding of the tetrameric K^+^ channel protein (Renart et al., 2020). On a microscopic level, PS specifically interacts with the KcsA protein, resulting in an elevated unitary conductance and open probability (Marius et al., 2008; Alvis et al., 2003; Iwamoto and Oiki, 2013). These lipid/protein interactions occur with individual cationic amino acids in a small helix upstream of the first transmembrane domain (Iwamoto and Oiki, 2013) and via structural motifs such as clefts between adjacent monomers (Renart et al., 2020).

**Figure 1.**
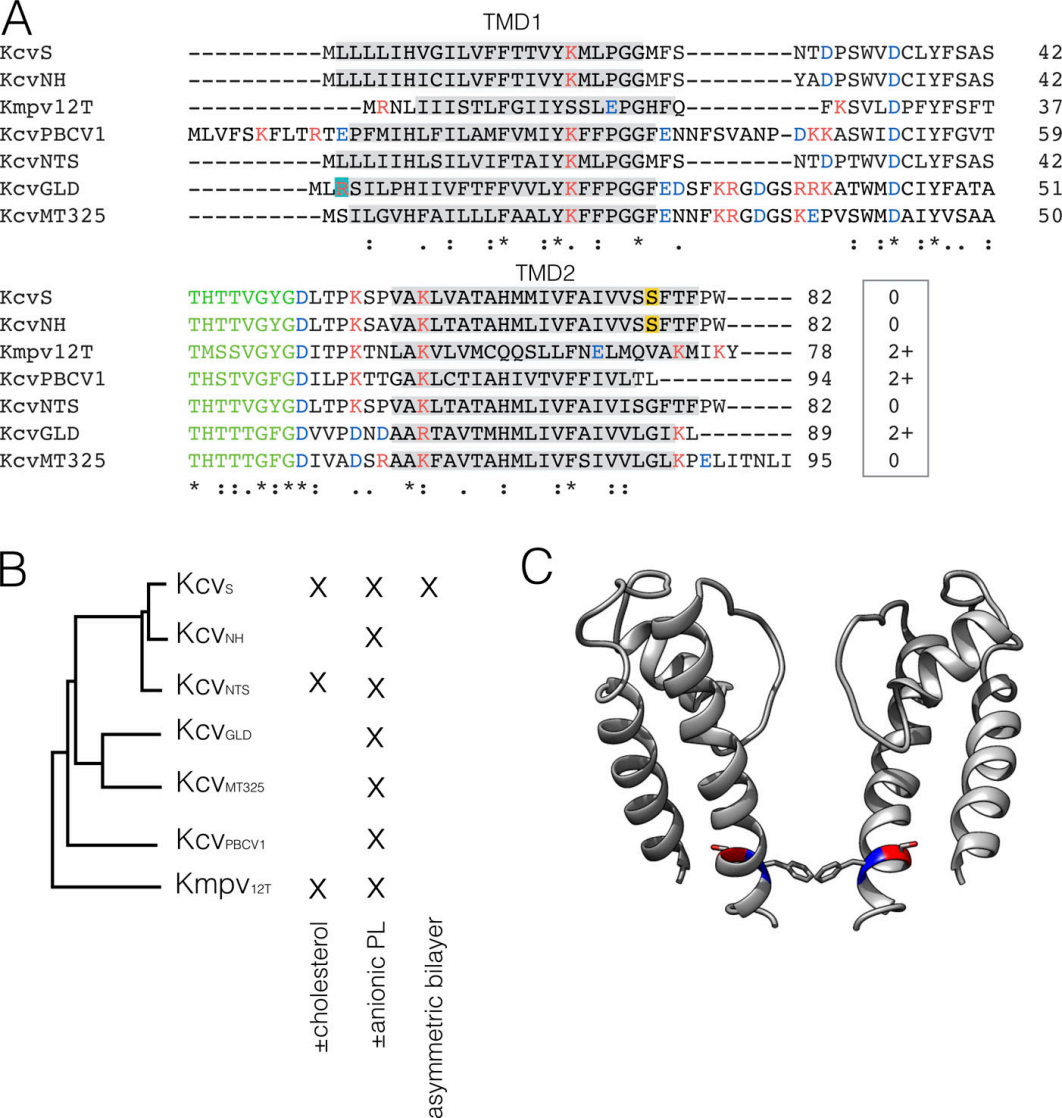
**Structural diversity of small viral K^+^ channels.** **(A)** Multiple sequence alignment of seven K^+^ channels encoded by different viruses in the family Phycodnaviridae*.* The monomers differ in size between 78 (Kmpv_12T_) and 95 amino acids (Kcv_MT325_). The conserved filter domain of K^+^ channels is shown in green. The predicted positions of the two transmembrane domains (TMD1 and TMD2) are marked in gray. Cationic (red) and anionic (blue) amino acids are highlighted. The critical amino acid Arg3 in Kcv_GLD_ is highlighted in turquoise, and the critical Ser for cytosolic gate in Kcv_S_ and Kcv_NH_ (see C) is highlighted in yellow. Numbers in the box give net charges of proteins. **(B)** Phylogenetic tree generated from data in A. The Xs indicate the types of experiments for which the channels were employed. **(C)** Model of Kcv_S_ channel with critical amino acids Ser77 (in red) and Phe78 (in blue). For clarity, only two of the four monomers in the tetrameric channel are shown.

From these data, we conclude that different types of protein/bilayer interactions are important for the function of ion channels. In light of the complex and dynamic composition of membranes, it is important to consider the lipid/protein interplay and to determine the rules that dictate these interactions. In the present study, we employ the natural diversity of seven small viral encoded K^+^ channels, together with their robust function in planar lipid bilayers, to investigate the interplay between channel proteins and the lipid bilayer. The small viral K^+^ channels are an excellent experimental system for a systematic analysis of functional interactions between channel proteins and lipid bilayers. The K^+^ channel proteins can easily be reconstituted for single-channel recordings in planar lipid bilayers with defined and even asymmetric compositions (Winterstein et al., 2018). Furthermore, the combination of in vitro translation into nanodiscs (NDs) and subsequent reconstitution in defined host bilayers uncouples potential effects of lipids on the folding of the protein (Renart et al., 2020) from the acute impact of PLs on channel function (Winterstein et al., 2018). The tendency of the Kcv channels to incorporate with a side-specific preference into the membrane and their distinct voltage dependency also make it easy to determine their orientation in the bilayer (Winterstein et al., 2018; Rondelli et al., 2018). A further experimental benefit is the presence of a large library of channels that all share the same Kir type architecture but differ at the same time in primary amino acid sequence, gating, and unitary conductance (Kang et al., 2004; Siotto et al., 2014; Rauh et al., 2017a; Murry et al., 2020). Most of the channels are very small with no cytosolic N or C termini (Fig. 1); in MD simulations, we found that they are barely long enough to span a 1,2-dimyristoyl-*sn*-glycero-3-phosphocholine (DMPC) or 1-palmitoyl-2-oleoyl-*sn*-glycero-3-phosphocholine (POPC) bilayer (Braun et al., 2014). We reasoned that the small size and the absence of cytosolic domains should make these proteins very sensitive to the bilayer composition. Also, all functional properties, which are influenced by the bilayer, should be directly mediated by the transmembrane domains or the amino acids at the membrane/water interface.

By functional reconstitution of the model channels in defined bilayers, we found that saturating concentrations of cholesterol have no appreciable impact on channel conductance and gating on two model channels. This finding suggests that cholesterol has no a priori impact on channel function and that the protein/bilayer system must have efficient ways of compensating hydrophobic mismatches; the latter is expected to occur with an increase in bilayer thickness in cholesterol-containing membranes. The effects of the sterol, which were reported for other channels (Levitan et al., 2010), must hence be mediated by specific binding of the sterol to the channel proteins.

Anionic PLs in all tested channels, irrespective of their structural diversity, generated an increase in unitary conductance. This general effect was further amplified by anionic PLs (aPLs) in the inner leaflet of the membrane in combination with cationic amino acids in the channel protein. aPLs also affected gating in some, but not all, channels. This suggests a sequence-dependent interaction between negatively charged lipids and channels proteins. In the case of two channels, which have a gate at the entry to their cavity, the negative PLs suppressed a defined closed state, which was determined by this gate. Hence, negatively charged PLs may affect the orientation of the transmembrane domains in such a way that this gate with its intrahelical hydrogen bonds is not functional.

## Materials and methods

### Planar lipid bilayer experiments

**Figure S1.**
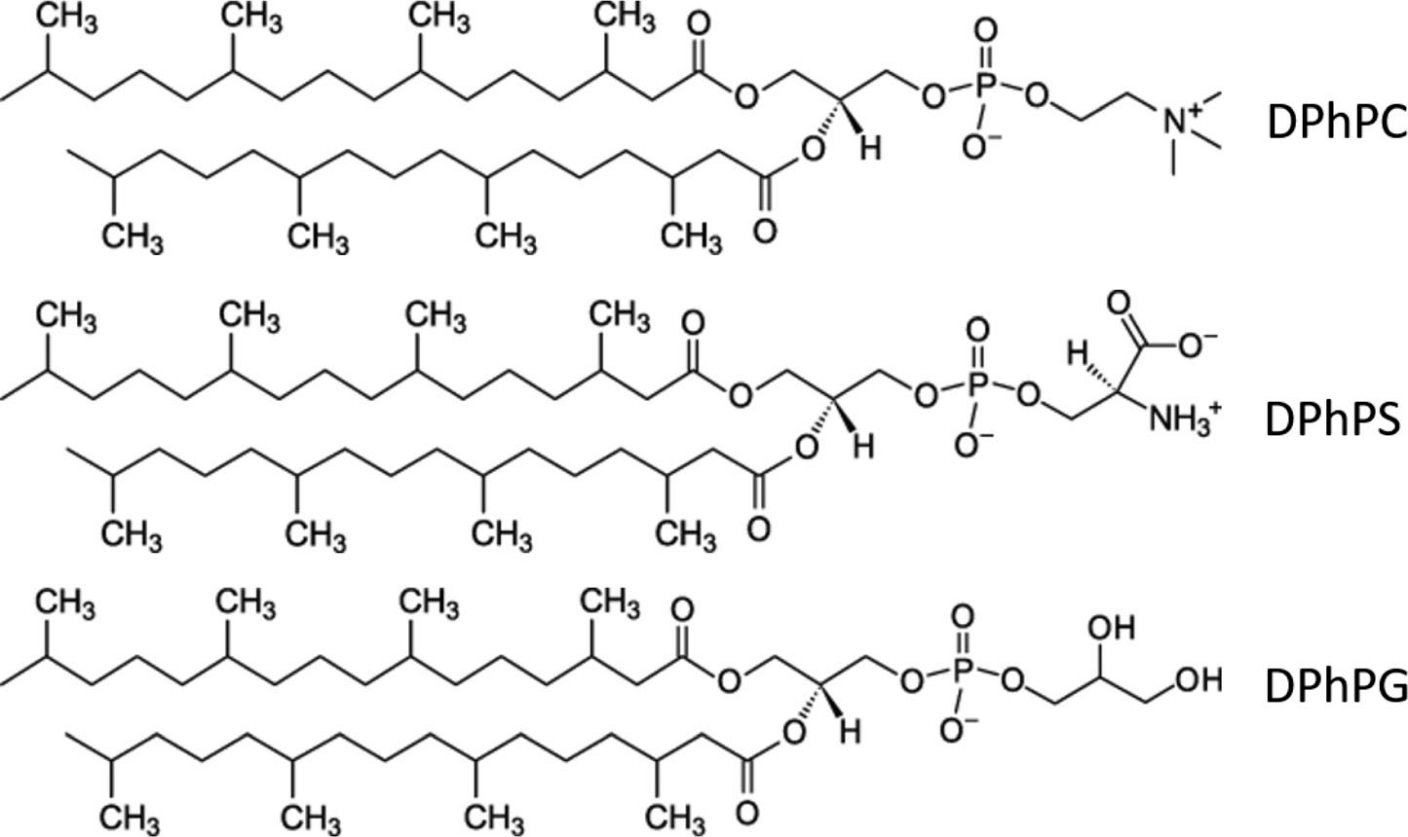
**Chemical structure of phospholipids used in this study.** DPhPC, DPhPS, and DPhPG.

Planar lipid bilayer experiments were done with a vertical bilayer setup (IonoVation) as described previously (Braun et al., 2014). A 1% hexadecane solution (Merck KGaA) in n-hexane (Carl Roth) was used for pretreating the Teflon foil (Goodfellow GmbH). The hexadecane solution (ca. 0.5 µl) was added to the rim of the hole (100 µm in diameter) in the Teflon foil with a bent Hamilton syringe. The experimental solution contained 100 mM KCl and was buffered to pH 7.0 with 10 mM HEPES/KOH. For bilayer formation, we used the following lipids from Avanti Polar Lipids (Fig. S1): 1,2-diphythanoyl-*sn*-glycero-3-phosphocholine (DPhPC), 1,2-diphythanoyl-*sn*-glycerol-3 phosphatidyl-serine (DPhPS), or n1,2-diphythanoyl-*sn*-glycero-3-phospho-(1′-rac-glycerol) (DPhPG). Cholesterol (Avanti Polar Lipids) was added to some DPhPC bilayers at a concentration of 0.15–25 mg/ml in n-pentane (Merck KGaA). Ion channel fluctuations were recorded with an EPC7 amplifier (List Medical) and digitized at 5 kHz after 1-kHz filtering with the LIH 1600 from HEKA Electronic. Data were analyzed with KielPatch (version 3.20 ZBM/2011) and Patchmaster (HEKA Electronic).

### Contact bubble lipid bilayer experiments

Contact bubble lipid bilayer experiments were used to measure channel activity in asymmetric bilayers (Iwamoto and Oiki, 2015). Two borosilicate glass pipettes (Tube, Capillary, melting point, 1.5–1.8 × 100 mm; Kimble Chase) with a tip diameter of ≤30 µm were pulled with a micropipette puller (PP-830) and filled with measuring solution (100 mM potassium chloride and 10 mM HEPES, pH 7). The solution in both pipettes also contained liposomes of DPhPC or DPhPS as well as channel proteins in NDs. We typically added 2–8 µl of a 1:10 dilution from the first elution fraction of the in vitro synthesis/purification to 25 µl of the pipette solution (Winterstein et al., 2018). A low concentration of the channel/ND preparation increases the chance of single-channel recordings. The glass pipettes were mounted on micromanipulators, and their tips were immersed in a hexadecane bath. Upon applying pressure to both pipettes, small bubbles (<50 µm diameter) formed at their tips in the oil–water interface. By maneuvering the glass pipettes, the two bubbles were brought in close contact, which resulted in the formation of a small bilayer at the contact zone with a capacitance of 2–5 pF in <1 min. Following bilayer formation, channel proteins from the pipette solution inserted spontaneously into the host membrane. Their activity was measured via Ag/AgCl electrodes in the pipettes with an amplifier (3900A Integrating Patch Clamp, Dagan). Currents were filtered at 1 kHz and digitized with a sampling frequency of 10 kHz by an A/D converter (Instrutech LIH 8+8 data acquisition system; HEKA Electronic).

#### Liposome preparation

To produce liposomes, 15 mg of the desired lipid (Avanti Polar Lipids Inc.) was solubilized in 1 ml chloroform (AppliChem), dried in a glass vial, and finally resuspended in 10 ml measuring solution. Subsequently, the lipid solution was sonicated at room temperature for a minimum of 1 h until it was transparent. After liposome preparation, the solution was frozen in 50-µl aliquots and thawed for further use.

#### Protein production

All channels were synthesized cell free according to the manufacturer’s instructions (MembraneMax HN Protein Expression Kit; Invitrogen), as reported previously (Winterstein et al., 2018). In vivo synthesis occurred on a shaker with 1,000 rpm at 37°C for 1.5 h in the presence of NDs with DMPC lipids. The scaffold proteins of the NDs were His-tagged, which allowed purification of channel/ND complexes via metal chelate affinity chromatography. The concentration of His-tagged NDs in the reaction mixture was adjusted to 30 µM. For purification of the channel/ND complexes, the crude reaction mixture was adjusted to 400 µl with equilibration buffer (10 mM imidazole, 300 mM KCl, and 20 mM NaH_2_PO_4_, pH 7.4, with KOH) and then loaded on an equilibrated 0.2-ml HisPur nickel-nitrilotriacetic acid agarose (Ni-NTA) spin column (Thermo Fisher Scientific). For binding of His-tagged NDs to the Ni-NTA resin, the columns were incubated for 45 min at room temperature and 200 rpm on an orbital shaker. In the subsequent step, the buffer was removed by centrifugation. To eliminate unspecific binders, the column was washed three times with 400 µl of a 20-mM imidazole solution. Finally, the His-tagged NDs were eluted in three fractions with 200 µl of a 250-mM imidazole solution. All centrifugation steps were performed at 700× *g* for 2 min. After purification, the elutions were stored at 4°C. For reconstitution of channel proteins into the lipid bilayer, a small amount (∼2 µl) of the purified channel/ND conjugates (dilutions) was added directly below the bilayer in the trans compartment.

#### Ion channel recordings, data analysis, and statistics

After insertion of an active channel into the bilayer, the membrane was routinely clamped for periods between 10 s and 15 min to a range of positive and negative voltages (usually from +160 mV to −160 mV in 20-mV steps).

Slow gating events (between the open state, O, and the “slow” closed states C1, C2, and C3 in Fig. 3 D, below) were detected by a Hinkley detector (Schultze and Draber, 1993) and then evaluated by dwell-time analysis.

Open-time histograms were fitted with one exponential function in order to calculate the mean open lifetime, τ_O_. Closed-time histograms were fitted with two or three exponential functions to obtain the mean closed time, τ*_Ci_*, and the number of closed events, *N_Ci_*, of each population of closed events. In cases where it could not be clearly decided by visual inspection of the histograms whether two or three exponential functions should be used, both fits were performed and the adjusted coefficient of determination (*R_adj_*^2^) was calculated:

$$R_{adj}^{2}=1-(1-R^{2})\frac{n-1}{n-p-1},$$ (1)

with *R*^2^ being the coefficient of determination, *n* the number of data points, and *p* the number of free variables of the used model function. The fit that provided the larger *R_adj_*^2^ value was then used. If the mean lifetime of long-lived and underrepresented closed states could not be determined by fitting, it was estimated by averaging the remaining closed events. To determine which events should be used for this calculation, we defined a threshold value. This corresponded to the dwell time for which the fit function predicted less than one event in the following bin of the dwell-time histogram. The resulting number of closed events and the calculated mean lifetime were subsequently treated as if they had been determined by fitting the histogram with an additional exponential function. Each population of closed times was then interpreted as an individual closed state, *Ci*, that can be reached exclusively from the open state, O, as shown in Fig. 3 D. This assumption allowed the calculation of the occupation probability, P_Ci_, and switching frequency, *f*_Ci_, of the closed state, *Ci*, as follows:

$$P_{Ci}=\frac{N_{Ci}.\tau_{Ci}}{N_{O}\;.\tau_{O}+\sum_{j=1}^{n}N_{Ci}.\tau_{Ci}}$$ (2)

and

$$f_{Ci}=\frac{P_{Ci}}{\tau_{Ci}},$$ (3)

with *N*_O_ being the total number of open events, τ_O_ the mean open lifetime, and *n* the number of closed-time populations. Because of the limited temporal resolution of the experimental setup, a notable number of submillisecond closed events were not detected. The underestimation of the number of closed events inevitably resulted in an overestimation of the mean open lifetime τ_O_. To obtain an approximation of the true mean open lifetime, a simple missed events correction was applied as described in Rauh et al. (2017a). Since only a single open state could be detected, the switching frequency, *f*_O_, of the open state is simply identical to the sum of the switching frequencies of all closed states.

In contrast to the slow gating transitions examined by dwell-time analysis, the open/close transitions of fast gating between the open state, O, and the fast closed states F and M (see Fig. 3 D, below) are smoothed by the low-pass filter of the recording setup and cannot be detected by a level detector. However, they cause “excess noise” (Heinemann and Sigworth, 1991). This excess noise results in broadening and asymmetry of the peaks in the amplitude histogram. If the gating can be described by a Markov model with one open and one closed state and the signal is filtered by a first-order low-pass filter (which is impractical for the experiments), then an analytical expression for the shape of the amplitude histogram is available (Yellen, 1984). In our experiments, we have to use a four-state Markov model of gating, and a Bessel filter of fourth order is employed. In this case, the amplitude histograms have to be generated by simulations. Two random generators determine the sink state and the time of a jump (both weighted by the assumed rate constant). The resulting signal is filtered by the adequate low-pass filter. From the resulting time series, the amplitude histogram is created. The final amplitude histogram is obtained by a convolution of this simulated amplitude histogram with the amplitude histogram of the setup noise.

A search algorithm modifies the assumed rate constants of gating in subsequent runs until the simulated amplitude histogram matches the measured histogram. Details are given in a recent article (Schroeder, 2015).

The dependency of the unitary conductance, G, and open probability, P_O_, on aPLs was quantified by fitting data in Fig. 5, D and E to a single saturating exponential function:

$$Y=Y_{max}\left(1-e^{-\frac{X}{k}}\right)+Y_{0},$$ (4)

where *Y_max_* is the maximal increase in conductance or P_O_, *Y*_0_ the conductance/P_O_ value in a pure DPhPC bilayer, and *k* the dose for half-maximal increase in conductance/P_O_.

The voltage dependency of the P_O_ of Kcv_NH_ S77G in DPhPC bilayers and of Kcv_NH_ in DPhPS bilayers was quantified by fitting the data in Fig. 8 E with the Boltzmann equation:

$$P_{O}=\frac{P_{O,max}-P_{O,min}}{1+\exp\left[z\frac{F}{RT}\left(V_{1/2}-V\right)\right]}+P_{O,min},$$ (5)

with P_O,_*_max_* being the maximal open probability, P_O,_*_min_* the minimal open probability, *z* the apparent valence, *F* the Faraday constant, *R* the gas constant, *T* the absolute temperature in Kelvin, and *V*_1/2_ the voltage at half-maximal P_O_.

Experimental data are generally presented as arithmetic/geometric mean ± arithmetic/geometric SD of *n* independent experiments. Statistical significances were evaluated by one-way ANOVA and Student’s *t* tests.

### Sequence alignment and structural predictions

Alignments and the phylogenetic tree were made with the Clustal omega algorithm (https://www.ebi.ac.uk/Tools/msa/clustalo). Transmembrane domains of all channels with the exception of Kmpv_12T_ were predicted by TMHMM (http://www.cbs.dtu.dk/services/TMHMM-2.0). The MINNOU (http://minnou.cchmc.org) algorithm was employed for Kmpv_12T_ because this channel has noncanonical TMD domains (Siotto et al., 2014).

### Online supplemental material

**Figure S2.**
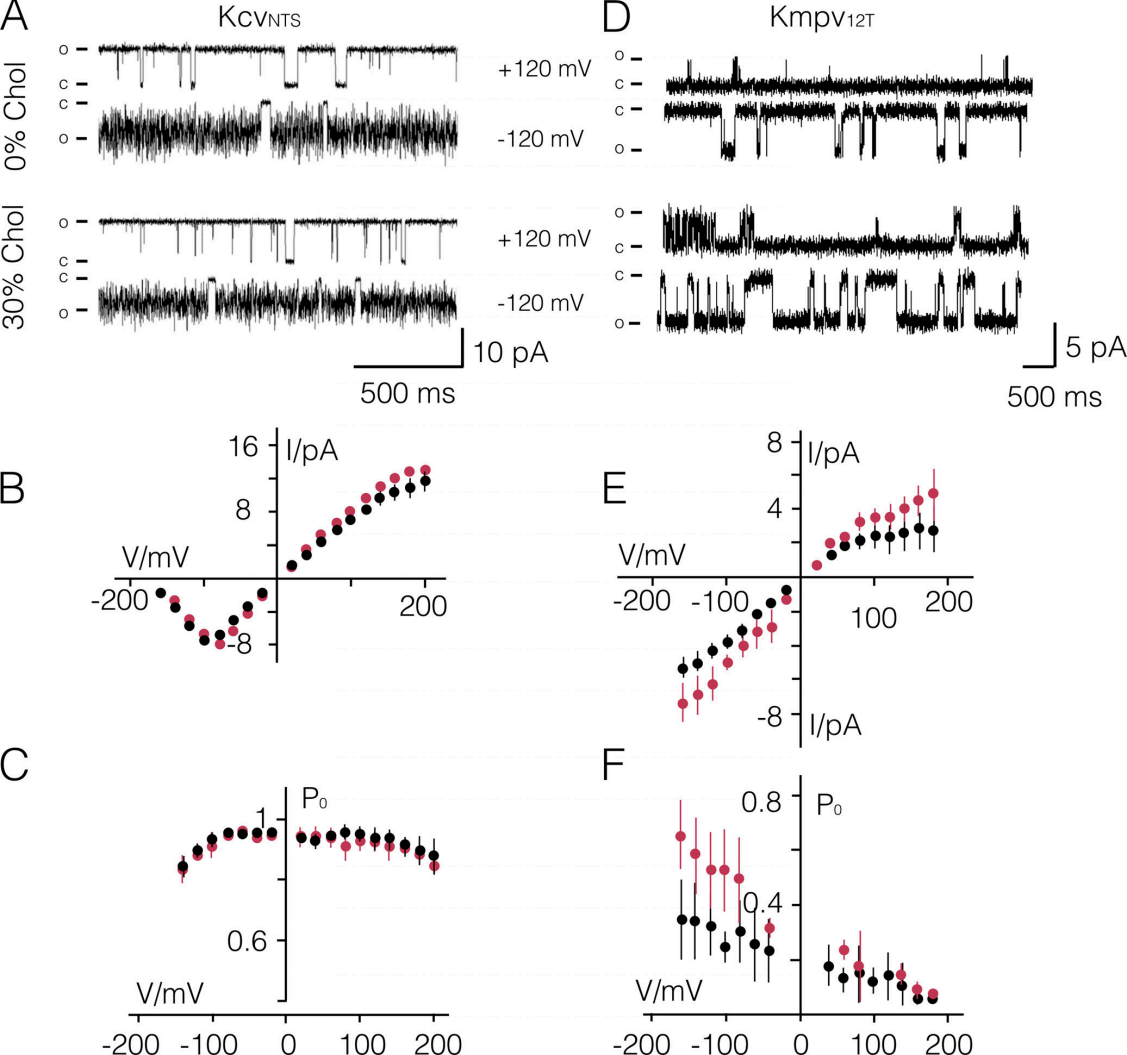
**Impact of cholesterol in phosphatidylcholine bilayers on Kcv_NTS_ and Kmpv_12T_ function.** **(A–F)** Representative fluctuations at ±120 mV in a pure DPhPC bilayer (0% Chol) and in DPhPC bilayer with 30 mol% cholesterol (30% Chol) from Kcv_NTS_ (A) and Kmpv_12T_ (D). Closed (C) and open (O) levels are indicated. I/V relations (B and E) and P_O_/V relations (C and F) of the respective channels on top in absence (black) and presence (red) of 30 mol% Chol. Data are mean ± SD of *n* = 6/4 and *n* = 10/3 measurements with Kcv_NTS_ and Kmpv_12T_ in the absence/presence of cholesterol, respectively. Chol, cholesterol.

**Figure S3.**
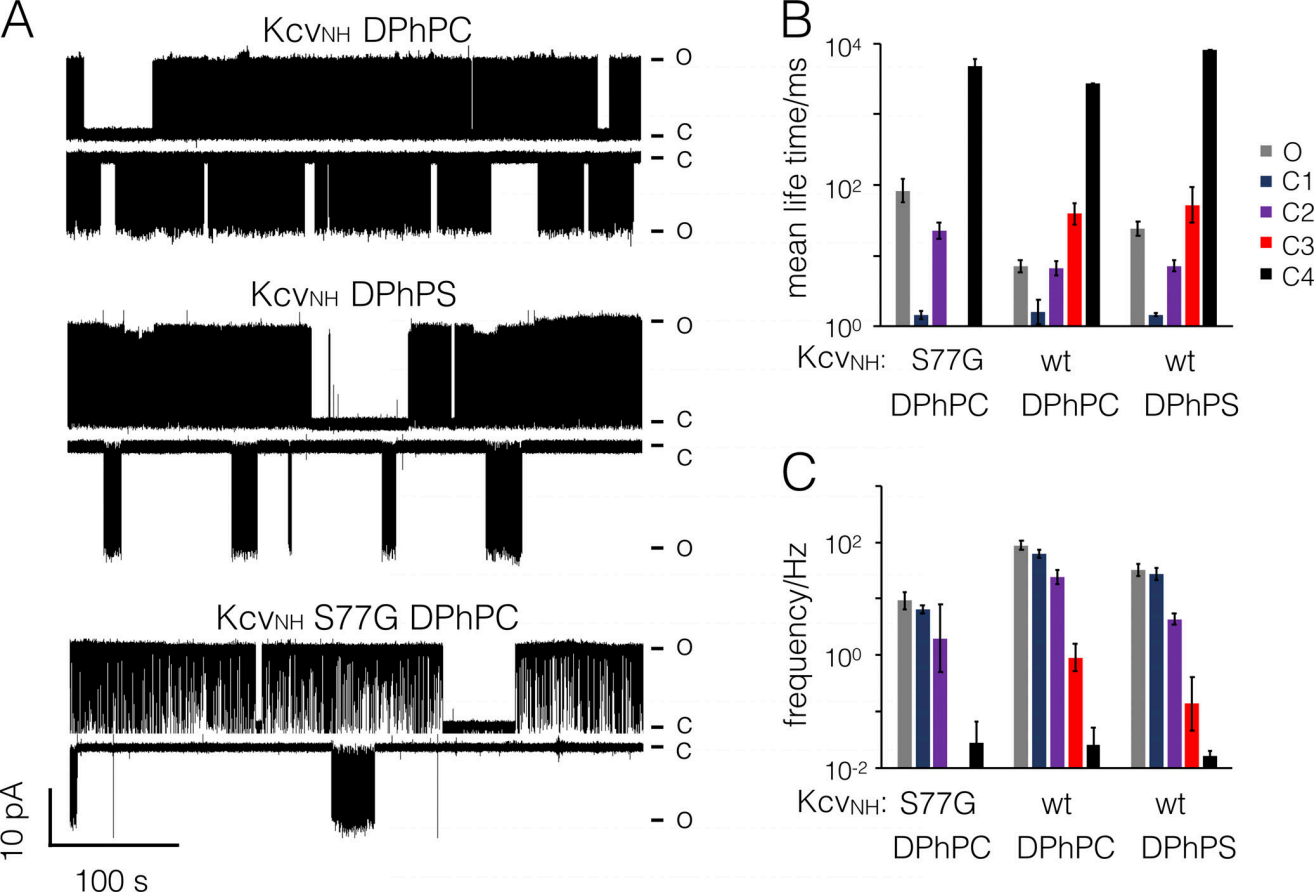
**Effect of aPLs on Kcv_NH_ and its mutant S77G.** **(A)** Representative single-channel fluctuations of Kcv_NH_-WT and Kcv_NH_S77G at ±120 mV in DPhPC bilayers and the same measurement of Kcv_NH_-WT in a DPhPS bilayer. Note the occurrence of ultra-long closed times at negative voltages of the mutant in DPhPC and WT channel in DPhPS bilayers. Closed (C) and open (O) levels are indicated. **(B and C)** Mean lifetime (B) and frequency of switching (C) into the respective states were extracted from dwell-time analysis in Fig. 8. Data are geometric means ± geometric SD from *n* = 3 independent experiments. wt, wild type.

Fig. S1 shows chemical structures of the phospholipids used in this study. Fig. S2 shows the impact of cholesterol in phosphatidylcholine bilayers on Kcv_NTS_ and Kmpv_12T_ function. Fig. S3 shows the effect of aPLs on Kcv_NH_ and its mutant S77G.

## Results

**Figure 2.**
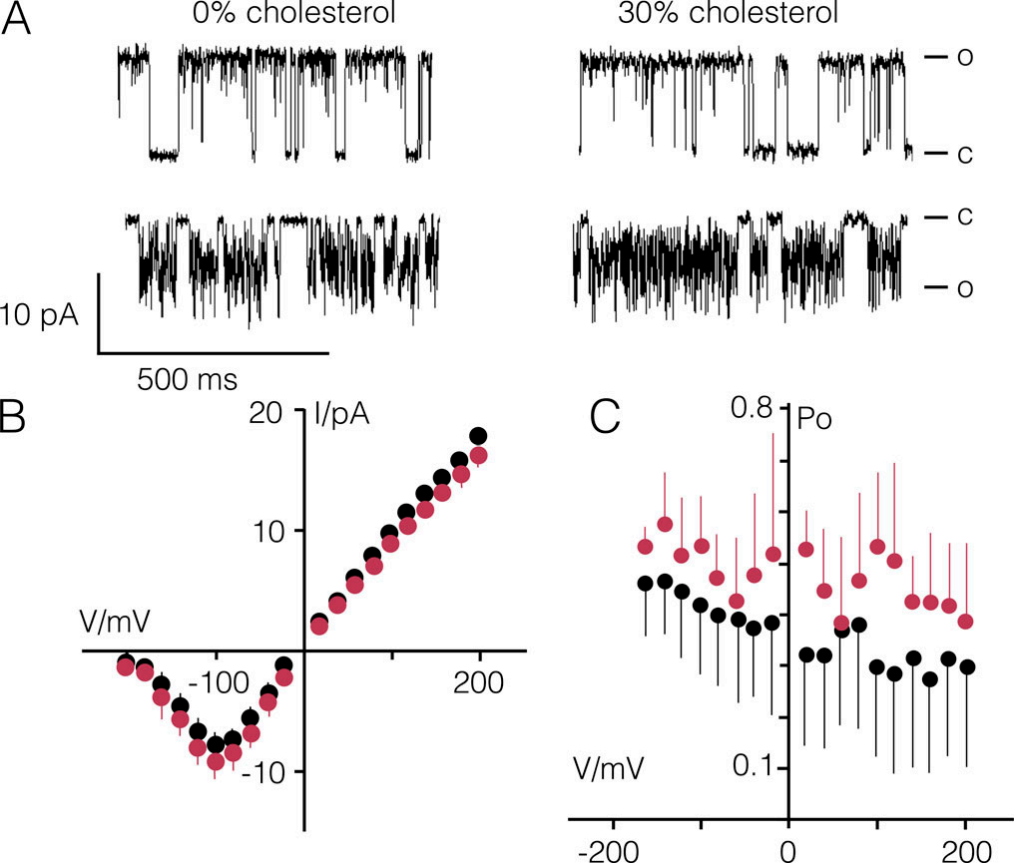
**Impact of cholesterol in phosphatidylcholine bilayers on Kcv_S_ function.** **(A)** Representative channel fluctuations of Kcv_S_ at +120 mV (top) and −120 mV (bottom) in a pure DPhPC bilayer (0% cholesterol) and in a DPhPC bilayer with 30 mol% cholesterol. Closed (C) and open (O) levels are indicated. **(B and C)** Open channel current/voltage (I/V) relations (B) and P_O_/V relations (C) in the absence (black) and presence of 30 mol% cholesterol (red). Data are mean ± SD of *n* = 10 (0% cholesterol) and *n* = 4 (30 mol% cholesterol) independent measurements.

To examine the effects of the bilayer composition on K^+^ channel activity, we chose Kcv_S_ (Fig. 1) as a reference channel because it has three well-defined closed and one open state that are detected by dwell-time analysis (Rauh et al., 2017a). In the first assays, we tested the dependency of Kcv_S_ gating on cholesterol in a phosphatidycholine bilayer. Fig. 2 A shows representative recordings of unitary channel fluctuations in a DPhPC membrane in the absence or presence of 30% cholesterol. The latter sterol concentration is typical for the plasma membrane of mammalian cells (van Meer et al., 2008). Detailed simulation studies of DMPC bilayers show that the impact of the sterol on structural parameters of the lipid membrane, such as bilayer thickness, approaches saturation at this concentration (de Meyer and Smit, 2009). Indirect evidence suggests a similar concentration dependency for DPhPC membranes in which the electrical stability, which serves as an indirect measure for membrane thickness, approaches saturation with 30% cholesterol (van Uitert et al., 2010).

In pure DPhPC membranes Kcv_S_ exhibits its typical functional features with clearly resolved channel fluctuations at positive voltages and a flicker type gating at negative voltages (Fig. 2 A). Unexpected for this small channel, which is barely long enough to span a PL bilayer (Braun et al., 2014), is that it is also active in a membrane with a high cholesterol concentration (Fig. 2 A).

The current/voltage (I/V) relations from *n* ≥ 4 recordings ± 30 mol% cholesterol show that the apparent unitary channel conductance is barely affected by the sterol (Fig. 2 B and Table 1). The visual impression that the sterol has no dramatic impact on the open probability is confirmed by open probability/voltage (P_O_/V) plots. The channel exhibits only a small increase in open probability with cholesterol (Fig. 2 C and Table 1), which lies within the large scatter of the measurements and should not be overinterpreted here.

**Table 1.** Effect of cholesterol on conductance (G) and open probability (P_O_) of channels ±30 mol% cholesterol (Chol) in DPhPC bilayers

Channel	Chol mol%	N	G/pS	P_O_ −100 mV
Kcv_S_	0	10	92 ± 9	0.40 ± 0.01
Kcv_S_	30	4	100 ± 12	0.53 ± 0.08
Kcv_NTS_	0	6	82 ± 12	0.93 ± 0.01
Kcv_NTS_	30	4	97 ± 8	0.91 ± 0.04
Kmpv_12T_	0	10	37 ± 5	0.21 ± 0.08
Kmpv_12T_	30	3	51 ± 9^a^	0.52 ± 0.16^a^

G measured as slope conductance between ±60 mV. Open probability is presented for each channel for the critical voltage of −100 mV at which a possible effect of cholesterol on P_O_ is most visible. Data are represented as mean ± SD of *n* independent bilayer recordings.

a The significance for a difference in G and P_O_ values ± Chol is indicated with P values <0.01.

The same experiment was conducted with Kcv_NTS_ and Kmpv_12T_. While the primary amino acid sequence of the former is very similar to Kcv_S_, the latter is only distantly related (Fig. 1, A and B). Both channels function in the absence and presence of 30 mol% cholesterol (Fig. S2, A and D). In the case of Kcv_NTS_, we observed a small positive effect on unitary conductance (Fig. S2 B), which is, however, within the scatter of the measurements. The sterol had no appreciable effect on the P_O_ value of this channel (Fig. S2 C and Table 1). However, since the P_O_ value of Kcv_NTS_ is already in the DPhPC bilayer close to 1, any positive cholesterol effect on this parameter may be concealed by the fact that it is already maximal. The general message of the experiments is that the two very similar Kcv proteins exhibit only small and no systematic sensitivities to a high cholesterol concentration, which implies that the sterol has no general impact on channel function.

Interesting to note is the positive effect of cholesterol on Kmpv_12T_ channel function (Fig. S2, D–F). This protein with only 78 amino acids per monomer is even shorter than the other Kcv type channels (Fig. 1 A). In the small Kmpv_12T_, the sterol causes a significant increase in unitary conductance as well as in the voltage-dependent open probability of this channel at negative voltages (Fig. S2, E and F; and Table 1). Collectively, these data confirm that a sterol concentration, which is typical for mammalian plasma membranes (van Meer et al., 2008), has only protein-specific and no general effects on functional parameters of K^+^ channels.

**Figure 3.**
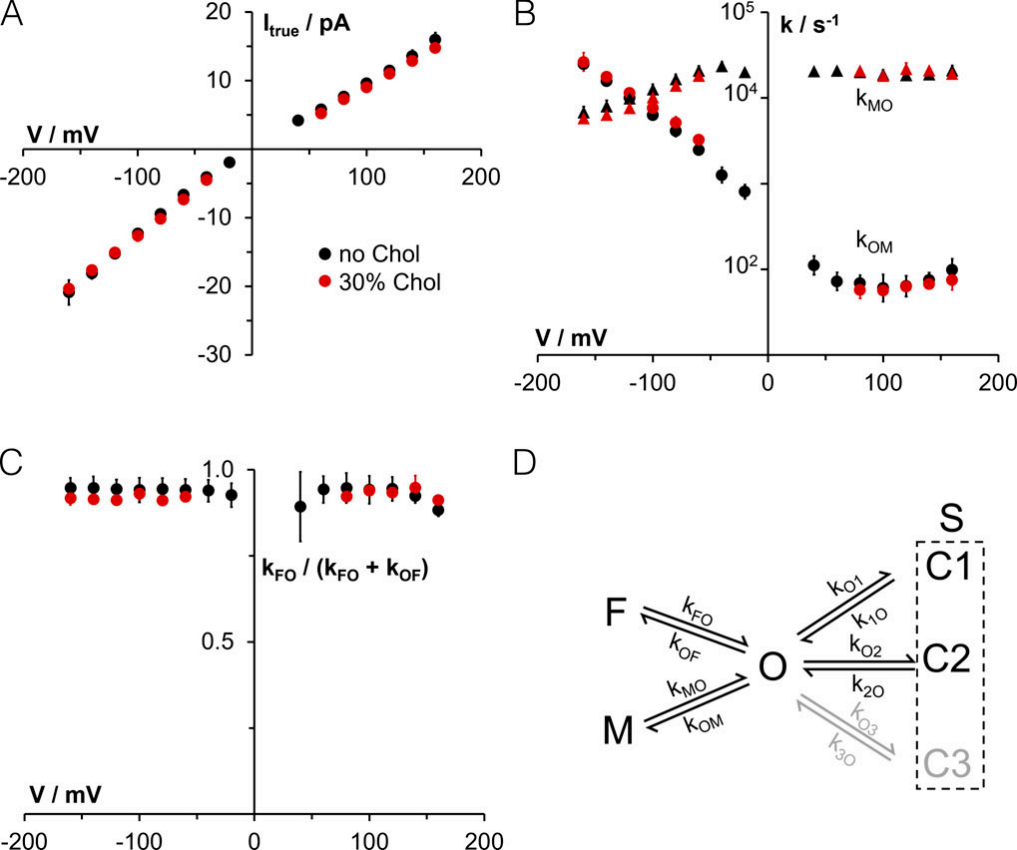
**Effect of 30 mol% cholesterol in DPhPC bilayers on the true current and fast gating in Kcv_NTS_ analyzed with β distributions.** **(A)** True single-channel current I*_true_.* **(B)** Rate constants of channel closing (k_OM_) and opening (k_MO_) of sub-millisecond gating (O-M gating). **(C)** Gating factor of the microsecond gating (O-F gating) k_FO_/(k_FO_ + k_OF_), i.e., the open probability of O-F gating. **(D)** Gating model used for analysis. The rate constants of gating between state O (open) and the short closed states F and M were analyzed with extended β distributions, the gating related to the longer closed states (C1, C2, and for Kcv_S_ additionally C3) with dwell-time analysis (see main text). Data points represent mean ± SD in A and C and geometric mean ± geometric SD in B. Six and three independent experiments were without and with cholesterol, respectively. At lower voltages, some data points are missing because the signal-to-noise ratio here was not sufficient for a reliable fit in all experiments. Chol, cholesterol.

Single-channel recordings of Kcv_S_ and Kcv_NTS_ further show that the sterol has no visible impact on the apparent decrease of conductance at negative voltages (Fig. 2, A and B; and Fig. S2, A and B), which is typical for Kcv channels and results from the fast voltage–dependent O-M gating (= the gating process between states O and M; Fig. 3, B and D) in the selectivity filter (Rauh et al., 2018). To test if cholesterol has some hidden effects on channel permeation and gating, which are fast and are not resolved as full open/closed transitions by the recording, we used an extended β distribution analysis of the well-characterized Kcv_NTS_. The true single-channel current (I_true_; Schroeder, 2015) and fast gating processes are extracted by fitting the amplitude histograms from current traces as in Fig. 2, A and B (Schroeder, 2015; Schroeder and Hansen, 2006). For these fits, the kinetic scheme of gating shown in Fig. 3 D was used for this channel; it includes one open state (O), two closed states of the fast gating processes (M = medium and F = fast) and two or three (depending on the Kcv isoform—the data in Fig. 3 refer only to Kcv_NTS_) slow gating states C1, C2, and C3, the latter three being detectable by dwell-time analysis (see below). Extended β distribution analysis cannot distinguish between these slow processes; therefore, they are merged into one representative state S for the analysis.

The data in Fig. 3 A show that the I_true_ of Kcv_NTS_ is not significantly affected by 30 mol% cholesterol in a DPhPC membrane. This underpins that the small difference in conductance ± cholesterol in Fig. S2 is an experimental inaccuracy rather than a physiological effect. Fig. 3 B further underscores the insensitivity of the rate constants k_OM_ and k_MO_ (Fig. 3 D) of the voltage-dependent O-M gating toward cholesterol. Also, the fast microsecond gating process (Fig. 3 C; O-F gating; Rauh et al., 2017b, 2018) of the open level in Fig. 2 A is not influenced by cholesterol. This is presented by the gating factor (open probability of the O-F gating: k_FO_/(k_FO_ + k_OF_; Fig. 3 D), because this gating process is at the edge of the temporal resolution.

The data confirm that the presence of cholesterol in the bilayer and its consequences on structural features of the bilayer have no appreciable impact on gating, including the fast gating in the filter. The absence of any significant effect of cholesterol on fast gating underscores the high structural stability of the selectivity filter. Collectively, the results of these experiments suggest that the channel must have an efficient strategy for avoiding any kind of hydrophobic mismatch, which is imposed by the presence or absence of cholesterol in the bilayer. The gates of the channel seem to be unaffected by this adaptation and the different physico-chemical flavors of the bilayer.

### Anionic lipids increase channel conductance

**Figure 4.**
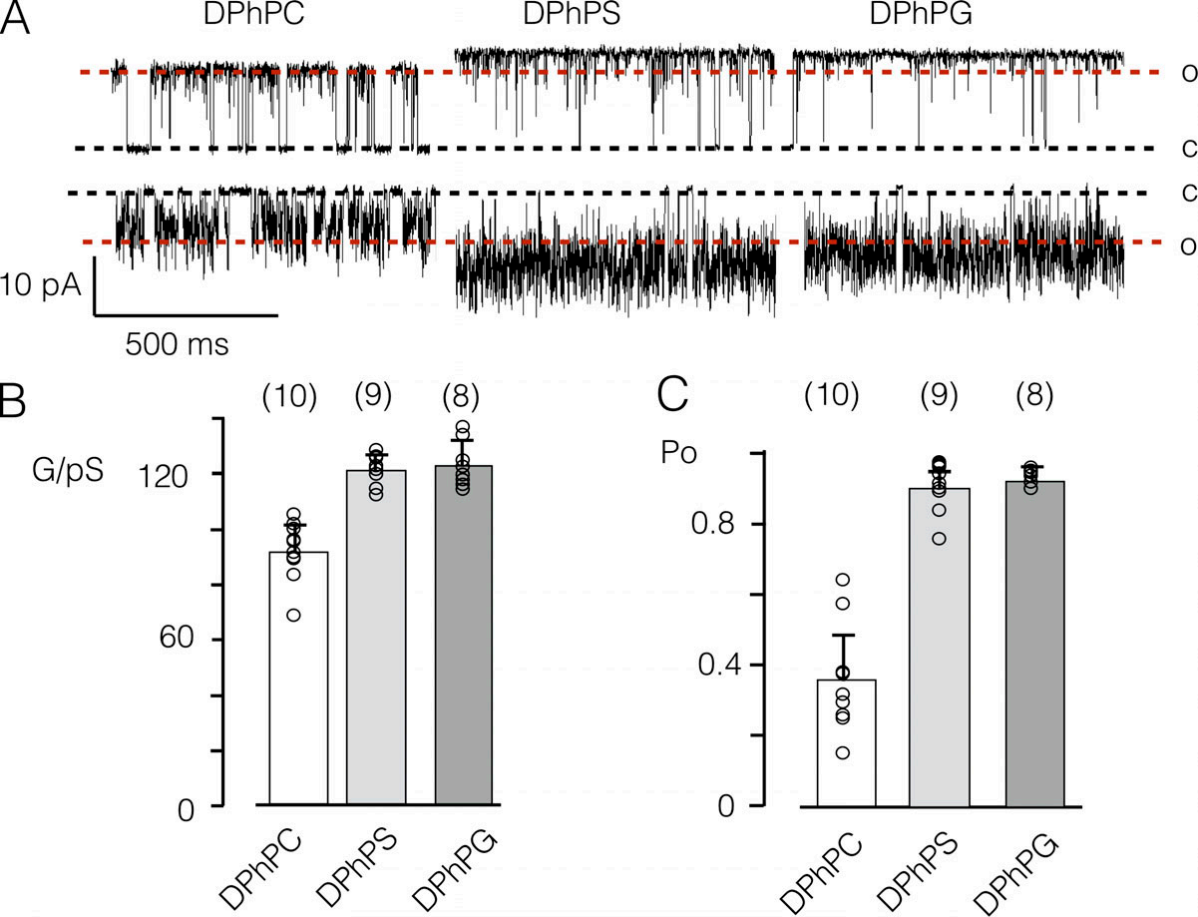
**Impact of aPLs on Kcv_S_ function.** **(A)** Representative channel fluctuations at +120 mV (top) and −120 mV (bottom) in DPhPC, DPhPS, or DPhPG bilayers. Closed (C) and open (O) levels are indicated. Dashed red lines mark the level of the open channel current in a DPhPC bilayer. **(B and C)** Mean conductance G (B) and mean open probability (C) of single-channel activity at +120 mV in the DPhPC, DPhPS, or DPhPG bilayer. Columns and error bars display means ± SD of number of independent measurements shown in brackets. The individual values are plotted as open black circles.

A number of reports imply that anionic lipids, which are present in appreciable concentrations in the plasma membrane (Rivel et al., 2019), have structural and functional implications for K^+^ channels (Marius et al., 2008; Alvis et al., 2003; Iwamoto and Oiki, 2013). To uncover general and protein-specific effects of these types of lipids, we measured the activity of Kcv_S_ in a membrane with a 1:1 mix of DPhPC and DPhPS. The latter has the same fatty acids scaffold of DPhPC but an anionic head group (Fig. S1). This negative charge has a profound impact on the functional features of the channel: it increases the unitary conductance and generates a strong elevation in open probability (Fig. 4). To test whether this stimulating effect of DPhPS is indeed caused by the negative charge or by other chemical peculiarities of the PL head groups, the experiments were repeated in a DPhPG bilayer; the latter also carries a negative charge but has a glycerol instead of a Ser as the head group (Fig. S1). Analysis of Kcv_S_ gating in a DPhPG bilayer provides values for unitary conductance and for the open probability, which are not distinguishable from those recorded in DPhPS (Fig. 4 and Table 2). The results of these experiments indicate that effects on the functional features of the channel are related solely to the anionic head group.

**Table 2.** Effect of aPLs on conductance (G) and open probability (P_O_) of K^+^ channels in lipid bilayers

Channel	Bilayer	N	G/pS	P_O_
Kcv_S_	PC	10	92 ± 9	−100 mV: 0.40 ± 0.10
Kcv_S_	PS	9	120 ± 6^a^	−100 mV: 0.89 ± 0.06^a^
Kcv_NTS_	PC	6	82 ± 12	−100 mV: 0.93 ± 0.01
Kcv_NTS_	PS	7	107 ± 5^a^	−100 mV: 0.97 ± 0.01^a^
Kcv_PBCV1_	PC	8	139 ± 11	+100 mV: 0.01 ± 0.004
Kcv_PBCV1_	PS	6	191 ± 11^a^	+100 mV: 0.12 ± 0.10^a^
Kcv_NH_	PC	3	98 ± 2	+60 mV: 0.47 ± 0.02
Kcv_NH_	PS	3	138 ± 4^a^	+60 mV: 0.72 ± 0.06^a^
Kcv_MT325_	PC	15	34 ± 6	+160 mV: 0.59 ± 0.14
Kcv_MT325_	PG	10	47 ± 6^a^	+160 mV: 0.70 ± 0.08
Kcv_GLD_	PC	7	38 ± 4	+160 mV: 0.72 ± 0.07
Kcv_GLD_	PG	9	91 ± 16^a^	+160 mV: 0.44 ± 0.05
Kmpv_12T_	PC	10	37 ± 5	−100 mV: 0.21 ± 0.08
Kmpv_12T_	PS	4	62 ± 4^a^	−100 mV: 0.10 ± 0.05

G measured as slope conductance between ± 60 mV. Open probability is presented for each channel for the indicated critical voltage at which a possible difference in P_O_ ± aPLs is most visible. Data are represented as mean ± SD of *n* independent bilayer recordings.

a The significance for a difference in G and P_O_ values for PC and PS/PG bilayers is indicated with P values <0.001.

**Figure 5.**
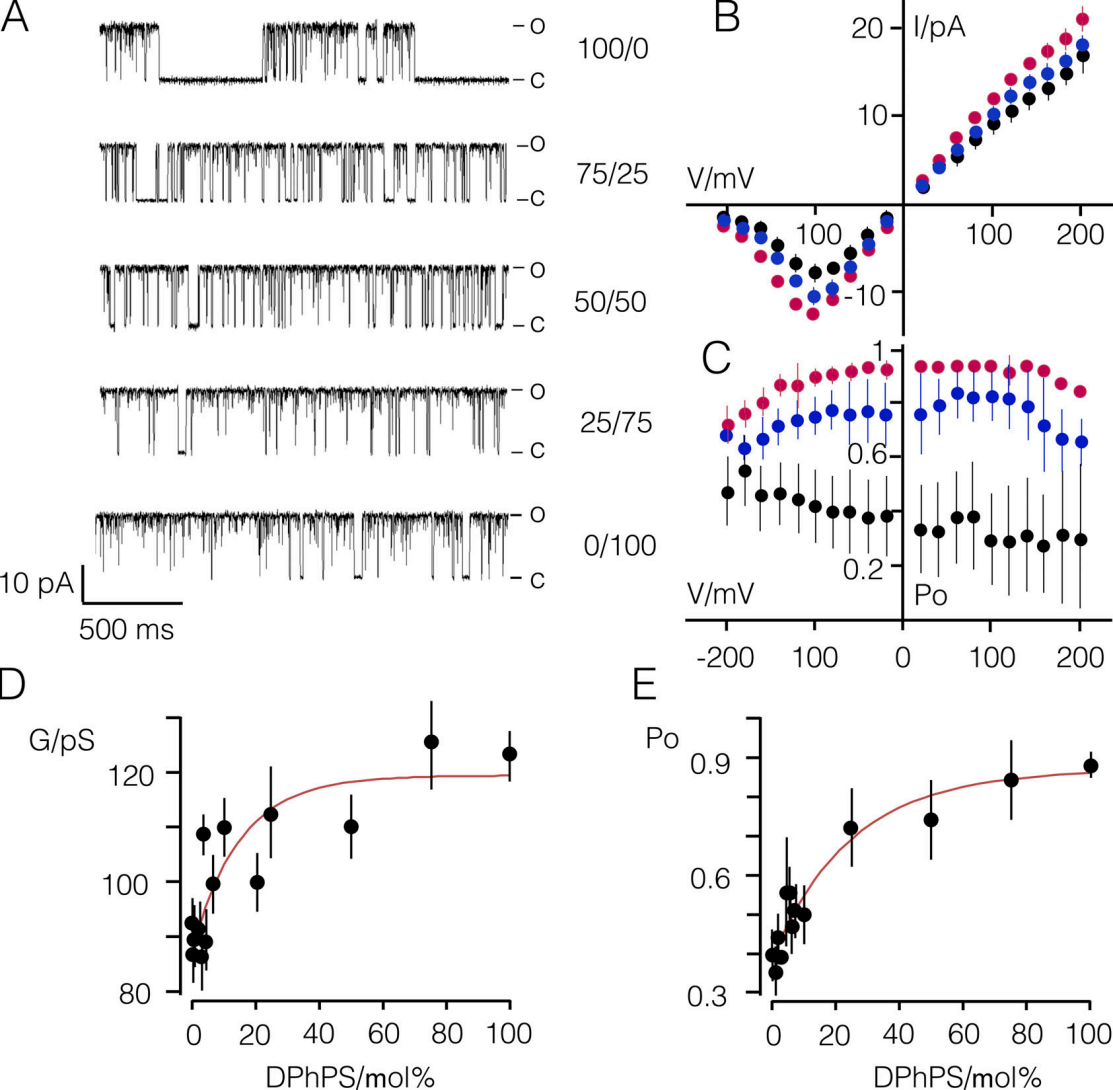
**aPLs increase Kcv_S_ conductance and open probability in a concentration-dependent manner.** **(A)** Representative single-channel fluctuations of Kcv_S_ at +120 mV in bilayers with an increasing content of DPhPS. Closed (C) and open (O) levels are indicated. **(B and C)** Mean single-channel I/V relations (B) and P_O_/V relations (C) for Kcv_S_ in DPhPC (black), DPhPS (red), and a 50:50 mix of DPhPC/DPhPS (blue) bilayers. **(D and E)** Mean conductance G (D) and P_O_ values (E) from experiments as in A–C as a function of DPhPS content (%) in the bilayer. Data in D and E were fitted with a single saturating exponential equation (Eq. 4) yielding a half-maximal DPhPS concentration for the stimulating effects of 15% (conductance) and 25.5% (P_O_), respectively. Data in B–E are mean values ± SD of *n* ≥ 5 independent experiments.

To further characterize this effect, the same experiments were performed over a range of DPhPS concentrations (Fig. 5 A). In a DPhPC bilayer, the channel exhibits its typical behavior with characteristic >100 ms–long closed times. As an immediate impression, it occurs that these closed events are suppressed with increasing concentrations of aPL. A more detailed analysis shows that the effect of the anionic lipid is more complex in that the unitary conductance and open probability increase with rising concentration of anionic lipid (Fig. 5, B–E). The dose/response curves can be fitted with a single saturating exponential equation (Eq. 4), yielding a 1.4- and 2.2-fold increase in conductance and open probability, respectively, between a pure DPhPC and a pure DPhPS bilayer. The half-maximal increase of both parameters is achieved by 15% (conductance) or 25% (P_O_) DPhPS in the membrane (Fig. 5, D and E).

### Impact of aPLs on the inner gate

It is well established that Kcv_S_ has three distinct closed and one open state (Fig. 3 D). The long-lived close time C3 (Fig. 3 D) is determined by a gate at the entrance to the cavity (Rauh et al., 2017a). This inner gate is formed by intrahelical Ser-mediated hydrogen bonds in the inner transmembrane helices, which flip an aromatic amino acid in or out of the ionic pathway (Fig. 1 C).

**Figure 6.**
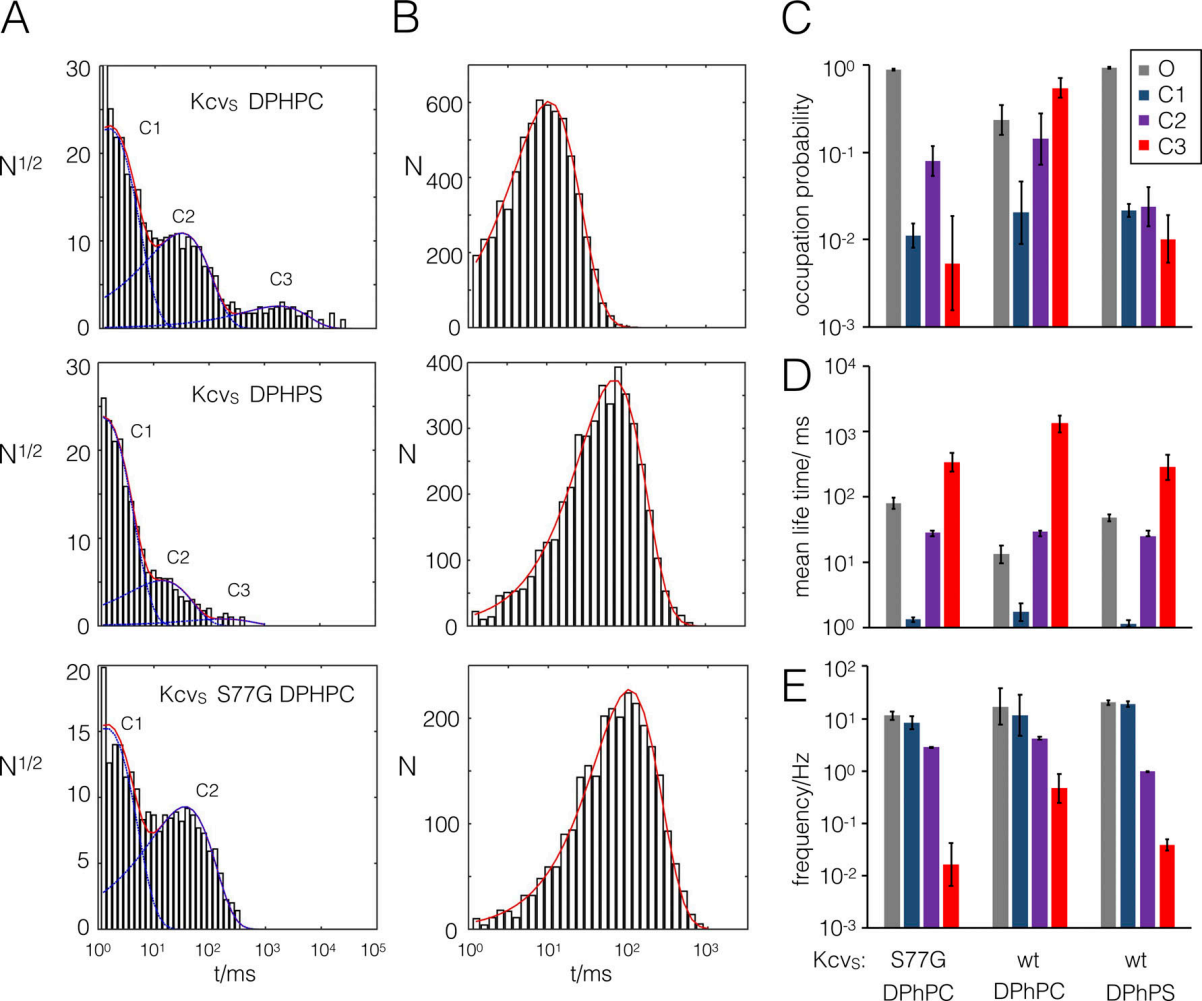
**Closed and open times of Kcv_S_ and Kcv_S_ S77G mutant in DPhPC and DPhPS bilayers.** **(A and B)** Exemplary closed (A) and open (B) times at +120 mV from Kcv_S_ and Kcvs S77G in DPhPC and DPhPS bilayers as indicated. Closed time data can be fitted with three or two exponentials (blue lines) for states C1–C3. The sum of these exponentials is shown as a red line. The open dwell time is in all conditions described by a single exponential (red line). **(C–E)** The mean occupation probability (C), mean lifetime (D), and frequency of switching (E) into the respective states were extracted from data in A and B. The assignment of the columns in C–E to the open state O and closed states C1–C3 is shown in the inset. Data are geometric means ± geometric SD from *n* ≥ 3 independent experiments. wt, wild-type.

To understand the mechanism by which aPLs elevate the open probability of Kcv_S_, we performed a comparative dwell-time analysis for measurements at +120 mV in DPhPC and DPhPS. In DPhPC, the channel exhibited the three expected closed states and one open state (Fig. 6, A and B). While these states are conserved in the DPhPS bilayer, the dwell-time analysis confirms the visual impression from Fig. 5 A that the occupation probability of the long-lived closed state C3 is strongly reduced in aPLs (Fig. 6, A and C). This reduction is mainly due to a decrease in the frequency with which the channel visits C3 (Fig. 6 E) and only to a small extent to a decrease in the mean lifetime of C3 (Fig. 6 D). The data furthermore show that the increase in open probability is supported by an additional reduction in the occupation probability of C2 (Fig. 6 C) and by a stabilization of the open state (Fig. 6, B and D).

For an understanding of the mechanistic impact of aPLs, it is interesting to note that a reduction in the occupation of C3 and a stabilization of the open state is also achieved by eliminating the aforementioned inner gate of Kcv_S_. Notably, a mutation of the critical Ser into a Gly (Kcv_S_ S77G) eliminates this gate (Rauh et al., 2017a), with the result that the C3 state disappears and that the open state is stabilized (Fig. 6). The frequent or rare presence of this long-lived closed time in DPhPC and DPhPS, respectively, (Fig. 5 and Fig. 6) implies that this gate might be suppressed by aPLs. This hypothesis will be further examined below.

**Figure 7.**
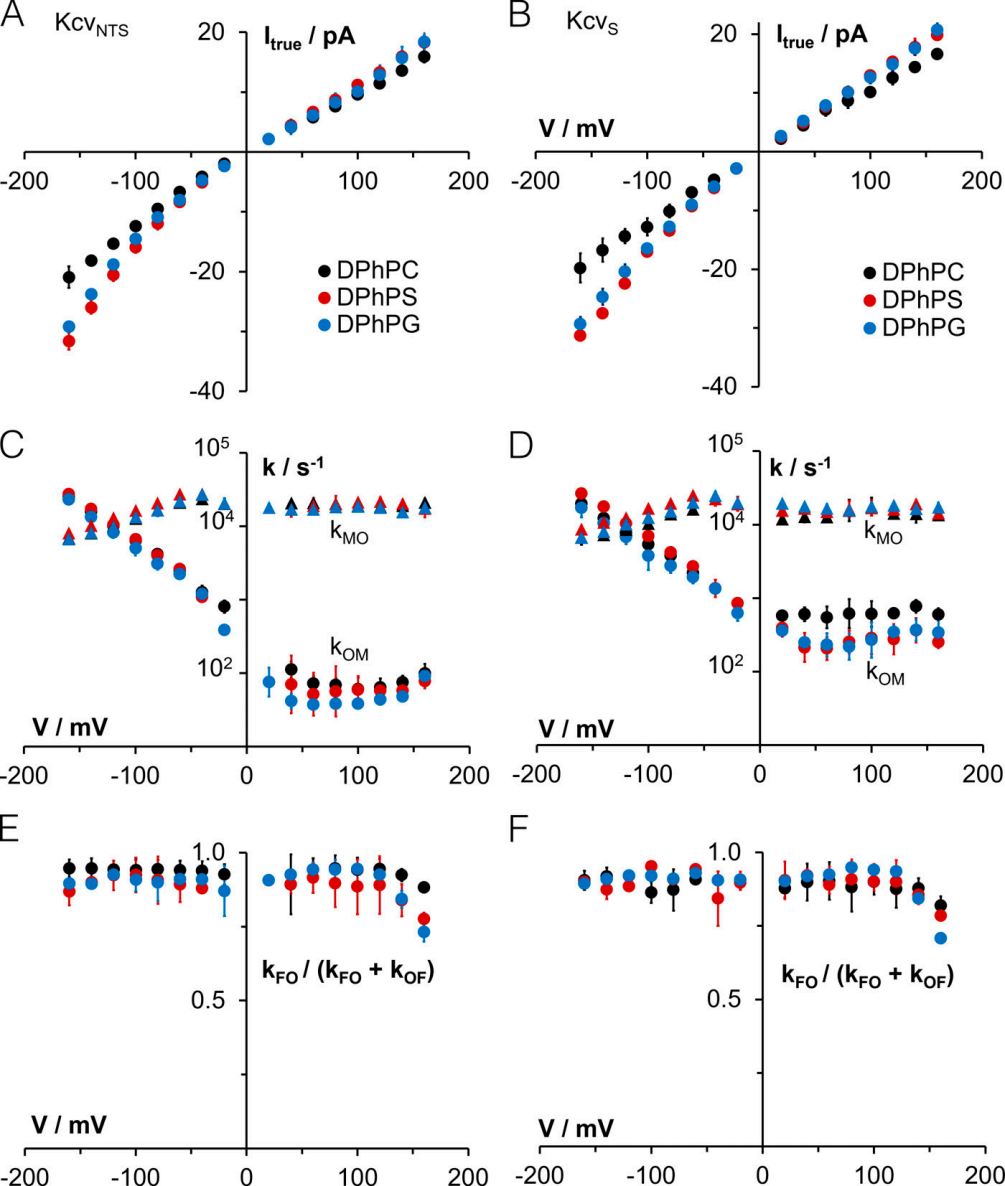
**Effects of the anionic lipids DPhPS and DPhPG on the true current and fast gating of Kcv_NTS_ and Kcv_S_ analyzed by β distributions.** **(A and B)** Effect on the I_true_ in Kcv_NTS_ and Kcv_S_. **(C and D)** Effect on the rate constants k_OM_ (closing) and k_MO_ (opening) of O-M gating (Fig. 3 D). **(E and F)** Effect on the gating factor of O-F gating (Fig. 3 D). Data points represent mean ± SD in A, B, E, and F and geometric mean ± geometric SD in C and D. Number of independent experiments: Kcv_NTS_ in DPhPC: *n* = 6; Kcv_S_ in DPhPC: *n* = 3, for +80 mV to +160 mV: *n* = 4; Kcv_NTS_ in DPhPG: *n* = 3, +40 mV to +120 mV: *n* = 4; Kcv_S_ in DPhPG: *n* = 3 at negative voltages, *n* = 6 at positive voltages; Kcv_NTS_ in DPhPS: *n* = 3; and Kcv_S_ in DPhPS: *n* = 3. At lower voltages, some data points are missing because the signal-to-noise ratio was not sufficient here for a reliable fit in all experiments.

In the next step, we analyzed the impact of aPLs on the filter gate. To examine whether the increase in apparent unitary channel current (Fig. 4 B and Fig. 5 B) indicates changes in I_true_ and to see whether this involves fast gating, we analyzed data from Kcv_S_ and Kcv_NTS_ with extended β distribution analysis (Schroeder, 2015). Fig. 7, A and B shows that the I_true_ indeed increases over the entire voltage window for both channels in anionic lipids. This increase is most pronounced at negative voltages, where it causes an exponential increase in the unitary current. This implies that the voltage-sensitive translocation step becomes rate limiting once the neutral reactions of ion loading or release increase.

The rate constants of O-M and O-F gating in neutral and anionic lipids are reported in Fig. 7, C–F. The rate constant of channel closing k_OM_ is insensitive to the anionic lipid at negative voltages, where it is determined by the voltage-sensitive ion occupation in the selectivity filter (Rauh et al., 2018). At positive voltages, a voltage-insensitive process dominates k_OM_ (Rauh et al., 2017b). This component is decreased by the anionic lipids in Kcv_S_ (Fig. 7 D) and also weakly in Kcv_NTS_ (Fig. 7 C). Since this component is sensitive to mutations in the cytosolic entrance (Rauh et al., 2017b), the data indicate that the anionic lipids do not affect the selectivity filter, but do affect the cytosolic entrance. The inverse reaction, k_MO_, is insensitive to the lipid. Worth noting is that we detected no significant effect of anionic lipids on O-F microsecond gating (Fig. 7, E and F) as presented by the “gating factor” k_FO_/(k_FO_ + k_OF_).

The slow gating processes between O and C1, C2, and C3 have identical influence on the shape of the amplitude histogram. Thus, the slow states are merged into a state S. The rate constants k_OS_ and k_SO_ are required for a good fit, but an assignment to the individual components is not possible. The sensitivity of these states to aPLs was extracted from the dwell-time histograms in Fig. 6.

### The anionic lipid suppresses a defined inner gate

**Figure 8.**
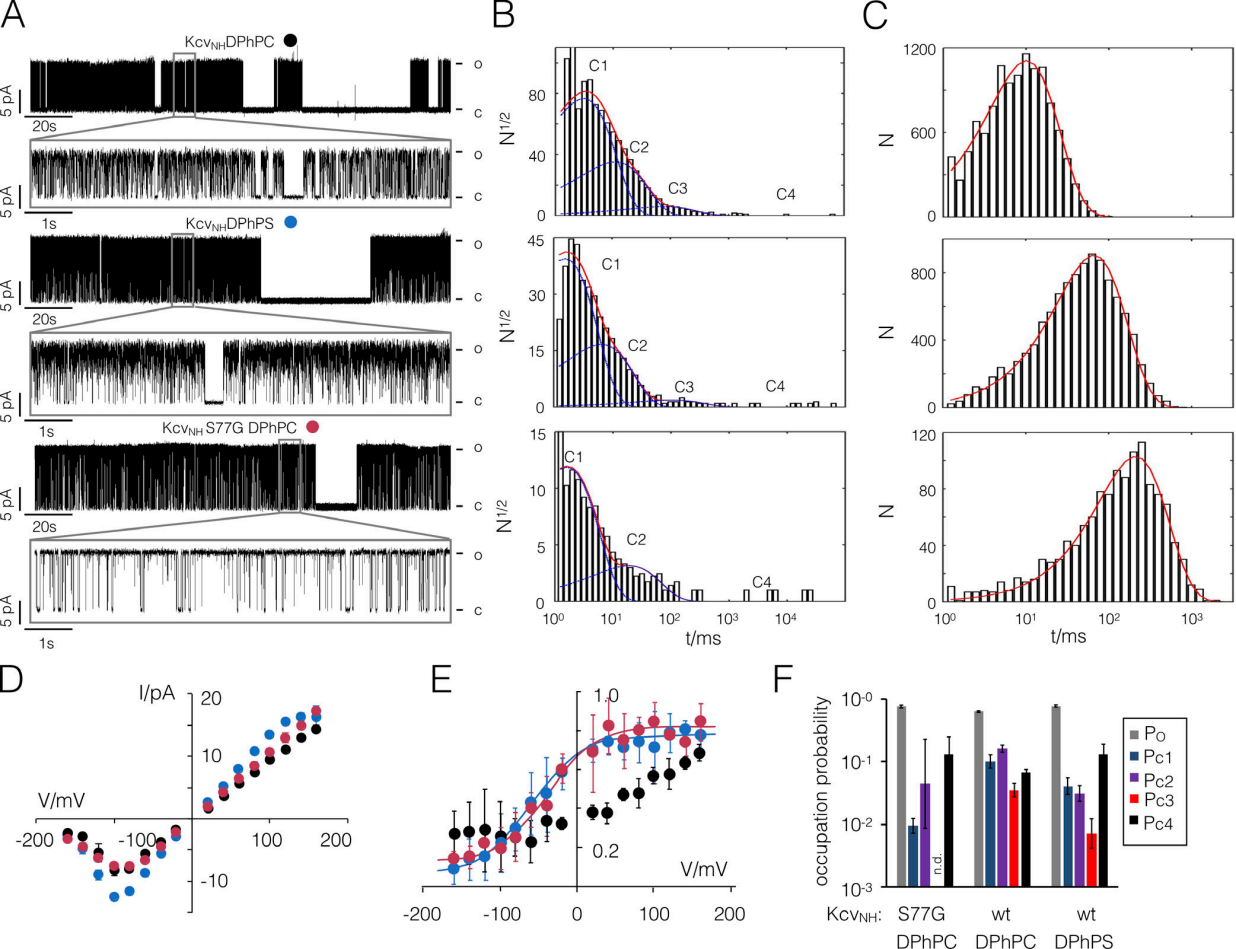
**Effect of aPLs on Kcv_NH_ channels.** **(A)** Representative single-channel fluctuations of Kcv_NH_-WT and Kcv_NH_ S77G at +120 mV in a DPhPC bilayer and the same measurement of Kcv_NH_-WT in a DPhPS bilayer. The sections indicated in the top rows are magnified below. Closed (C) and open (O) levels are indicated. **(B and C)** Exemplary closed (in B) and open (in C) dwell times recorded at +120 mV for Kcv_NH_ (top) and Kcv_NH_ S77G (bottom) in DPhPC bilayers as well as for Kcv_NH_ in DPhPS bilayers (center). The open state can be fitted by a single exponential (red line), while the closed times require three (top and center) or two (bottom) exponentials (blue line) for the different closed states C1–C3. The sum of these exponentials is shown as a red line). The mean lifetime of the small number of ultra-long–lived closed events from C4 has been estimated by calculating the arithmetic mean of the corresponding dwell times. Data are geometric means ± geometric SD from *n* = 3 independent dwell-time analyses. **(D and E)** Mean single-channel I/V relations (D) and P_O_/V relations (E) for channel recordings as in A. The symbols in D and E correspond to symbols in A. Data from E were fitted with a Boltzmann function (Eq. 5) yielding values for half-maximal activation at −37 ± 26 mV for the mutant in DPhPC and −57 ± 32 mV for the WT channel in DPhPS, respectively. **(F)** Occupation probabilities of open state P_O_ and four closed states P_C1–_P_C4_ for Kcv_NH_ and its S77G mutant in DPhPC and DPhPS bilayers. Data were extracted from dwell-time histograms as shown in B and C. The assignment of the column colors to the different states is given in the inset. Data points in D and E represent arithmetic mean ± SD of three experiments. Data in F represent geometric mean ± geometric SD of three experiments. wt, wild type.

The comparative dwell-time analysis of Kcv_S_ gating ± aPL implies a sensitivity of its inner gate to the charged PL. We tested this prediction by measuring the activity of another Kcv type channel (Kcv_NH_) that, like Kcv_s_, has a Ser at critical position 77 in the inner transmembrane helix (Fig. 1 A; i.e., the amino acid that forms the critical hydrogen bonds; Rauh et al., 2017a). After functional reconstitution of Kcv_NH_ in a DPhPC bilayer, this channel exhibits an overall low open probability, which increases at positive voltages (Fig. 8 E, black). The mutation S77G in Kcv_NH_, which mimics the amino acid composition of Kcv_NTS_ and eliminates the inner gate (Rauh et al., 2017a), also causes, as expected, an increase in open probability in this channel (Fig. 8, A and D; and Table 2). After elimination of the inner gate, the resulting P_O_/V curve exhibits the shape of an outward rectifier (Fig. 8 E, red) because an additional voltage-dependent gate, with an ultra-long–lived closed state C4 becomes dominant at negative voltages (Fig. S3 A). The P_O_/V plot of the mutant channel can be fitted with a Boltzmann function yielding values of −37 mV and 0.8 for the voltage at half-maximal P_O_ and for the valence *z*, respectively (Fig. 8 E).

Without going into details on the complex gating in this mutant, we predict that the same phenotype, which was generated by removing the inner gate, would also be achieved by recording the WT Kcv_NH_ channel in an anionic lipid. The data in Fig. 8, A–D show that this is indeed the case. Measurements of Kcv_NH_ activity in a DPhPS bilayer showed that this lipid increases not only the unitary conductance (Fig. 8, A and D, blue) but more importantly, the anionic lipid also mimics the effect that the S77G mutation has on gating. In DPhPS bilayers, the WT channel exhibits similar voltage-dependent P_O_/V characteristics as the mutant (Fig. 8 E and Fig. S3); the Boltzmann fit yields values of −57 mV and 0.8 for the voltage at half-maximal P_O_ and for *z*. The results of these experiments confirm the hypothesis that anionic lipids are able to modulate the closed state of a distinct gate, which is formed by intrahelical hydrogen bonds and bending of the inner transmembrane helix. This assumption is further supported by a dwell-time analysis (Fig. 8, B, C, and F).

While the WT channel exhibits the typical three closed states C1, C2, and C3 and a small number of ultra-long–lived closed events, which were assigned to the not fully resolved closed time population C4, the long-lived state C3 is no longer visible in the S77G mutant. In recordings of the WT channel in aPLs, the C3 state is still visible but strongly suppressed, suggesting that the charged PLs inhibit the closing of this gate. This is more evident from the analysis of the occupation probabilities (Fig. 8 F) and switching frequencies (Fig. S3 C) of the different states. As in the case of Kcv_S_, the aPLs as well as the S77G mutation reduce the occupation probability of C3 by nearly one order of magnitude. This goes together with a stabilization of the open state (Fig. 8 C and Fig. S3 B) and a reduction in the occupation of C2 (Fig. 6 and Fig. 8). These results suggest that the anionic lipids affect channel gating by interacting with the intracellular pore entrance. To further test this hypothesis, we performed single-channel measurements in asymmetric lipid bilayers.

### Anionic lipids act from the inner leaflet of the membrane

**Figure 9.**
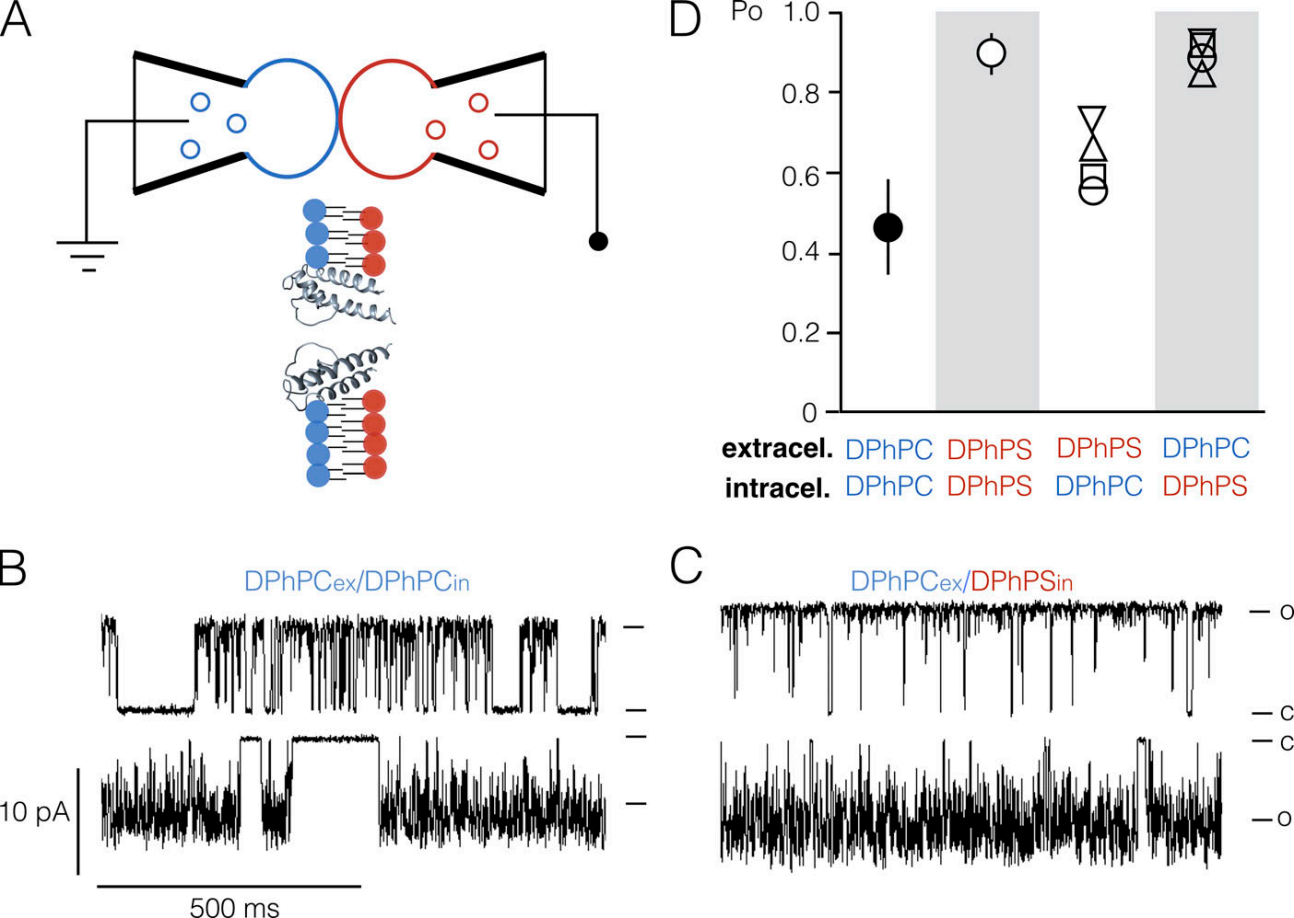
**Recording of Kcv_S_ in asymmetrical bilayers.** **(A)** Principle of the contact bubble bilayer method. Glass pipettes filled with liposomes of different lipids (red and blue circles) are immersed in an oil bath. By applying pressure to the pipettes PL monolayer bubbles with different lipid (red or blue), compositions are produced in the oil bath. At the contact point, the two monolayers form a bilayer in which the two leaflets are made from different PLs. Blowup: After insertion of the channel protein into the asymmetric bilayer, the cytosolic and extracellular sides will be exposed to different PLs. **(B and C)** Representative channel fluctuations at +120 mV (top) and −120 mV (bottom) in a symmetrical DPhPC bilayer (in B) and in an asymmetrical bilayer with DPhPS on the cytosolic side of the channel (in C). Closed (C) and open (O) levels are indicated. **(D)** Mean P_O_ values for single-channel recordings of Kcv_S_ in symmetrical or asymmetrical bilayers with DPhPC and DPhPS on the cytosolic or external side. Data are mean values ± SD (*n* = 4) for recordings in symmetrical bilayers and values from individual measurements (triangels, square, circle) for asymmetrical bilayers.

In the case of KcsA, it was found that anionic lipids affect channel function more in the inner than in the outer leaflet of the membrane (Iwamoto and Oiki, 2013). The results discussed above also imply that gating of the Kcv channels Kcv_S_ and Kcv_NH_ is mainly affected by interactions of anionic lipids with the cytosolic side of the protein. To test this hypothesis, we fabricated asymmetrical bilayers using the contact bubble bilayer method (Fig. 9 A), which allows the anionic lipid to exist in a side-specific manner relative to the Kcv_S_ channel. Control experiments in which Kcv_S_ was reconstituted with this method in symmetrical DPhPC or DPhPS membranes confirmed the general sensitivity of the channel to the different lipids (Fig. 9, B and D). In DPhPS, we see the expected increase in open probability over that in DPhPC (Fig. 9 D).

Next, Kcv_S_ channel activity was recorded in an asymmetrical bilayer in which the leaflet facing the side from which the channel proteins enter contains DPhPC. From previous experiments, it is well established that Kcv channels insert into the membrane with a strict bias (Winterstein et al., 2018; Rondelli et al., 2018). In the present experimental setting, the extracellular side of the channel was exposed to the leaflet consisting of DPhPC while the cytosolic side was facing the leaflet made of DPhPS. The representative channel recordings in Fig. 9 C show that Kcv_S_ exhibited the typical flicker gating at negative voltages in this setting. This confirms that the protein is indeed oriented in the membrane so that the cytosolic side of the membrane is exposed to DPhPS lipids. In this orientation, the channel acquires an elevated open probability and increased unitary conductance. The same results were obtained in four experiments with this orientation of the channel in asymmetrical bilayers (Fig. 9, C and D). In experiments in which the channel inserted with the cytosolic side oriented toward the DPhPC leaflet, it mostly maintained a lower open probability (Fig. 9 D).

At this point, we cannot answer the question of why the P_O_ value in the asymmetrical measurements with DPhPS on the extracellular side is higher than in symmetrical DPhPC bilayers. In principle, this could originate from some mixing of the phospholipids during the bilayer formation. It could also indicate an additional effect of anionic lipids on the extracellular side of the bilayer. Collectively, the results of these experiments indicate a leaflet specificity of aPLs on channel gating. Anionic lipids interact with the protein on the cytosolic side of the bilayer leaflet.

### Effect of anionic lipids on different channels

**Figure 10.**
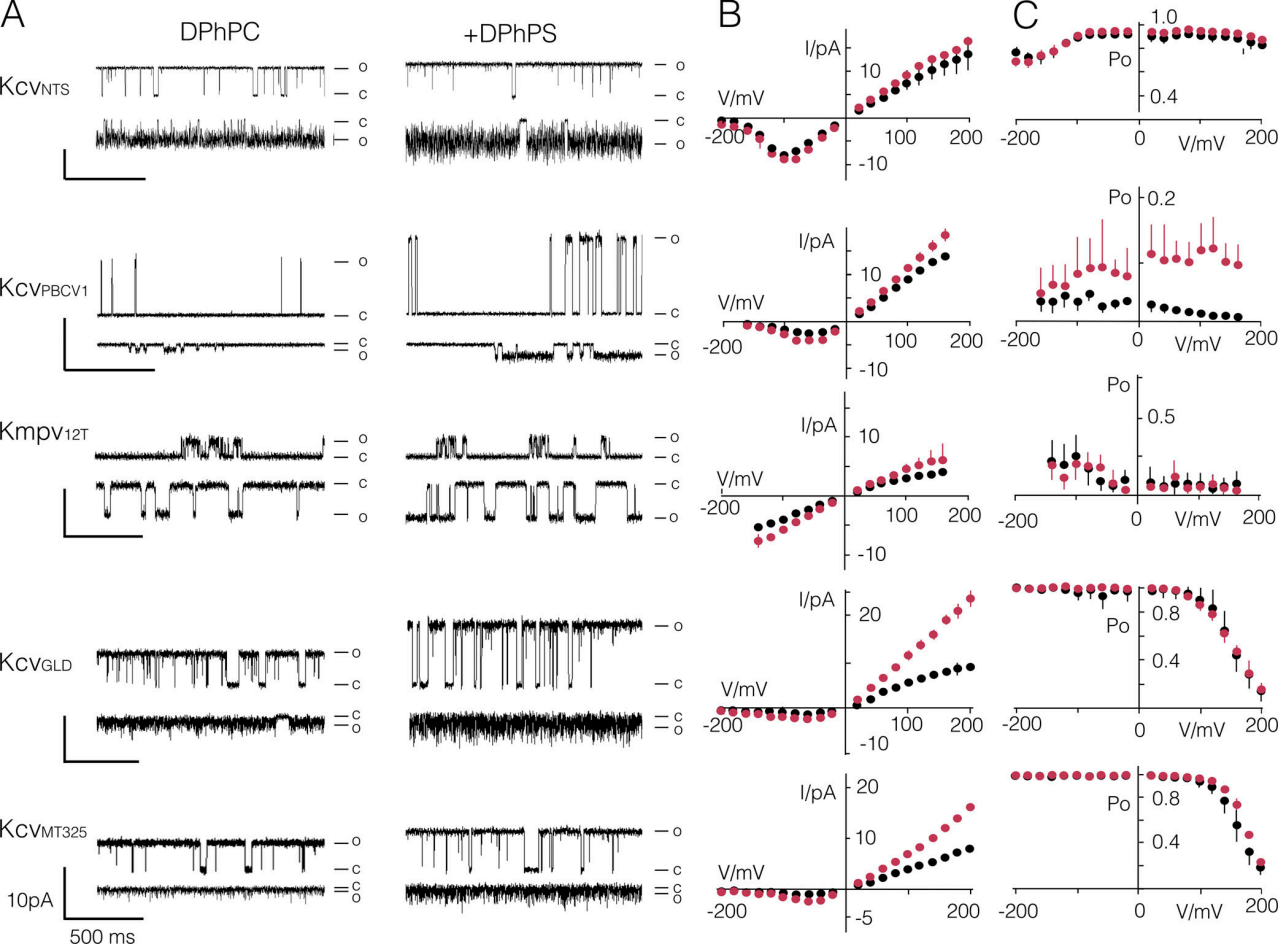
**Effect of aPLs on diverse viral K^+^ channels.** **(A)** Representative single-channel fluctuations of channels indicated on the left at ±120 mV in bilayers with only DPhPC or in bilayers containing a 1:1 mix of DPhPC:DPhPS. Closed (C) and open (O) levels are indicated. **(B and C)** Mean single-channel I/V relations (B) and P_O_/V relations (C) for channels in the same row from recordings in DPhPC (black) or DPhPC:DPhPS (red) bilayers. Data in B and C are mean values ± SD from *n* independent measurements of channel X in N_DPhPC_/N_DPhPC:DPhPS_. Kcv_NTS_: 6/7, Kcv_PBCV1_: 8/6, Kmpv_12T_: 10/8, Kcv_GLD_: 7/3, Kcv_MT325_: 5/3.

In further experiments, we addressed the question of whether the sensitivity of channel function to anionic lipids is specific to the similar Kcv_S_ and Kcv_NTS_ channels or is a general feature of small viral channels. Therefore, the experiments of Fig. 4 were repeated with five additional Kcv-type channels (Fig. 1 B). All channels are small proteins, which differ to variable degrees in their primary sequence and net charge (Fig. 1 B) as well as in their function from Kcv_S_; the latter is used here as a reference. A test of channel function in bilayers with either DPhPC or DPhPS shows that aPLs increased the unitary conductance in all the channels tested (Fig. 10 and Table 2). The positive effect on unitary conductance is dependent on the channel protein; it is small (1.2-fold) in Kcv_S_ but large (2.4-fold) in Kcv_GLD_.

The effect of aPLs on the open probability in contrast is more complex. It increases P_O_ not only in Kcv_S_ (Fig. 4 and Fig. 5) but also in Kcv_PBCV1_ over the entire voltage range (Fig. 10 C). In other channels the effect is more difficult to assess. In Kcv_NTS_, the P_O_ value is already close to 1 so that any potential positive effect on the open probability is difficult to resolve, considering that the channel is already close to maximally open. Also, in Kcv_GLD_ and Kcv_MT325_, the P_O_ value is close to 1 and only decreases at voltages >100 mV. In this voltage window, however, aPLs have no appreciable impact on the open probability of the two channels. Finally, in Kmpv_12T_, the overall low P_O_ value is not affected by the presence of aPLs (Fig. 10 C and Table 2). Collectively, these data suggest that aPLs have no common mode of impact on the open probability of different K^+^ channels.

In contrast to this, the data show that anionic lipids have in all cases a positive impact on unitary channel conductance. Because of the diversity in the primary structure and net charge between the channels (Fig. 1), this appears to be a general feature of the anionic lipid. However, the finding that aPLs stimulate the conductance of the channels to different degrees means that the interplay between lipids and channel must exhibit some amino acid sensitivity. While anionic lipids have a general positive effect on unitary conductance, they have variable impacts on P_O_. This means that anionic lipids presumably have multiple and independent impacts on a channel protein and that unitary conductance and P_O_ are not causally connected.

### Anionic lipids interact with cationic amino acids

**Figure 11.**
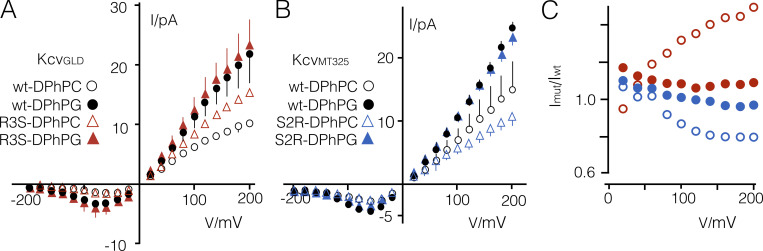
**Cationic amino acids in Kcv channels affect impact of aPLs on unitary channel conductance.** **(A and B)** Mean I/V relations of Kcv_GLD_ (A) and Kcv_MT325_ (B) and their mutants in DPhPC (open symbols) or DPhPG (closed symbols) bilayers. **(C)** Ratio of unitary current amplitudes from recordings of mutants (I_mut_; Kcv_GLD_-R3S [red] or Kcv_MT325_-S3R [blue]) divided by those of the respective WT channel (I_wt_) in DPhPC (open symbols) or DPhPG (closed symbols) bilayer. Data are mean ± SD from *n* ≥ 3 independent recordings. wt, wild type.

After identifying the importance of the inner leaflet of the membrane for channel function, we asked the question whether cationic amino acids on this side of the channel are important in this context. An interesting pair of channels to tackle this question is provided by the two similar channels Kcv_GLD_ and Kcv_MT325_ (Fig. 1 B). While Kcv_GLD_ has a cationic amino acid close to the N terminus, Kcv_MT325_ does not (Fig. 1 A). To examine whether Arg3 in Kcv_GLD_ contributes to its twofold higher sensitivity to aPLs compared with Kcv_MT325_ (Fig. 10 and Table 2), the cationic amino acid was mutated to Ser, which is the equivalent amino acid in Kcv_MT325_. Functional testing of the mutant in DPhPC shows that this mutation already causes a 1.5-fold increase in conductance (Fig. 11 A), suggesting that a positive charge in this region of the channel is suppressing unitary conductance. This positive effect of the arginine-to-serine mutation is further augmented by aPLs (Fig. 11 A). In DPhPG bilayers, the mutant reaches the same high conductance as the WT channel. This means that a positive charge on the cytosolic side of the channel has a negative effect on conductance only if the channels are embedded in neutral membranes. In negatively charged membranes, WT and mutant channels behave identically. Hence, elimination of the positive charge at the N terminus has no further positive effect on unitary conductance in negatively charged membranes.

To test whether the same effect also occurs in Kcv_MT325_, we performed the reverse experiment by introducing a cationic amino acid at the equivalent position in Kcv_MT325_. The functional data again verified the importance of a charge in this region of the channel (Fig. 11 B); the S2R mutation reduced unitary conductance in neutral PLs compared with the WT channel, confirming the negative impact of a positive charge in this position on conductance. In aPLs, the conductance increased to the same maximal level as in the WT channel, again underlining that both processes are not additive. The results of these experiments are compatible with the view that a positive charge in the protein at the protein/lipid interface has a negative effect on unitary channel conductance. This inhibitory impact is fully alleviated by aPLs.

Interestingly, the inhibitory effect of a positive charge in DPhPC bilayers is voltage dependent and increases with positive voltages (Fig. 11 C). This voltage-dependent inhibitory effect is eliminated by substituting the positively charged amino acid with Ser or by reconstituting the channels in aPLs. While neutralization of the amino acid and the aPL both increase the conductance, their modes of action must be different. The former only has a positive effect on conductance at positive voltages, while the latter increases conductance over the entire voltage range. This suggests that the positive charge of the amino acid near the N terminus specifically interacts with ions entering the cavity from the cytosol. This inhibitory impact is fully eliminated by the anionic headgroups of the lipid, presumably by charge neutralization.

## Discussion

Here, we employ seven small K^+^ channels with different degrees of structural and functional diversity to examine the impact of cholesterol and anionic lipids on basic channel functions. Since most of these channels have no cytosolic N or C termini, their functional dependencies on bilayer composition can be entirely interpreted in the context of an interplay between the transmembrane α-helices with their surrounding bilayers. From an analysis of single-channel recordings, we can form some general but also protein-specific insights into the importance of the bilayer on channel function.

One surprising finding was that a high concentration of cholesterol has no perceivable impact on conductance and gating of Kcv_S_ and Kcv_NTS_. Even more astonishing is that Kmpv_12T_, which is four amino acids shorter than Kcv_S_ and Kcv_NTS_, is not only functional in a cholesterol-containing bilayer but also exhibits a moderate increase in both functional parameters. These data are remarkable considering that the Kcv_NTS_ proteins appear in MD simulations barely long enough to span the thickness of a cholesterol-free POPC or DMPC bilayer (Braun et al., 2014). Hence, the structural impacts of a saturating concentration of the sterol on the membrane bilayer (de Meyer and Smit, 2009) has no effect on channel function. The insensitivity of the two Kcv channels rather suggests that the proteins must have efficient strategies for avoiding a hydrophobic mismatch between the transmembrane domains of the channel and the bilayer. This could include conformational changes in the protein and/or a deformation of the bilayer at the lipid–protein interface (Strandberg and Killian, 2003). Such a mismatch must become problematic for short channel proteins since a 30% sterol content in the bilayer should increase the thickness of the membrane by nearly 20% (de Meyer and Smit, 2009). The data do not provide any information on how the proteins adapt to the changing lipid environment. The only information that can be extracted from the present data is that this adaptation not only guarantees the general functionality of the channels but also conserves all their particular gating characteristics. This implies that the channel protein does not undergo any major conformational changes since the latter would have impacts on the channel gates.

Comparative analysis of the channels in bilayers ± anionic PLs uncovered general and protein-specific effects. A general feature of all seven channels was that their unitary conductance increased in membranes with anionic lipids. The fact that the same response was reported for KcsA (Marius et al., 2008; Alvis et al., 2003; Iwamoto and Oiki, 2013), a channel that shares little similarity in the primary amino acid sequence with the viral K^+^ channels (Plugge et al., 2000), supports the hypothesis that this reflects a general impact of anionic lipids on channel proteins. A detailed analysis of two of the viral channels (Kcv_S_ and Kcv_NTS_) revealed virtually no effects of aPLs on fast gating. This indicates that the increase in conductance is a structural and not a kinetic effect (i.e., aPLs do not decrease the velocity of otherwise unresolved fast closings in the open channels).

The structural variability between the different viral channels and KcsA suggests that the general positive effect of aPLs on their unitary channel conductance does not require a distinct binding motif for anionic lipids in these proteins. Also, the difference in net charges between the channel proteins (Table 1) provides no direct clue for a mechanistic explanation of this process. Taken together, the data suggest that anionic lipids must have some form of global effect on unitary conductance in K^+^ channels, which is not directly dependent on the protein. One mechanism that must be considered here is the tendency of anionic lipids to accumulate cations at the membrane/solvent interface (Kostritskii et al., 2016). It is possible that DPhPS/G–containing bilayers promote accumulation of K^+^ at the membrane/solvent boundary with a positive effect on unitary conductance. In this case, we would expect an effect of aPLs on fast gating in Kcv_NTS_, in which the rate constant k_OM_ (Fig. 3 D) proved to be very sensitive to the K^+^ concentration (Rauh et al., 2018). However, since our analysis shows that fast gating is insensitive to aPLs (Fig. 3 B), we must assume that an increase in local concentration of K^+^ at the boundary of aPL-containing bilayers is not significantly contributing to the increase in unitary conductance.

Close scrutiny of two similar channels, in which one has a high and the other a low sensitivity of its conductance to anionic lipids, uncovers the potential importance of cationic amino acids for sensitivity to anionic lipids. The data show that a cationic amino acid at the protein/water interface causes a voltage-dependent decrease in channel conductance. This inhibitory effect is alleviated by aPLs presumably by neutralizing the charge of the amino acid. This in turn could alter the function of the proteins and serve as a so-called electrostatic switch (Alexander et al., 2011; Jeffries et al., 2012; Tsuchiya et al., 2018).

The data also show a stimulating effect of anionic lipids on the open probability in some, but not all, channels. From these data, we can conclude that the effect of the charged lipids on gating is protein specific and is related to the primary amino acid sequence. For two of the channel candidates, we can provide a plausible molecular explanation for the stimulating effect of aPLs on the open probability. In a previous study (Rauh et al., 2017a), it was shown that a long-lived closed state in Kcv_S_ was determined by a gate at the entrance to the cavity. This gate is operated by an intrahelical hydrogen bond, which rotates an aromatic amino acid in or out of the ionic pathway. Here, we found that the presence of anionic lipids attenuated the critical long closed times in those two channel proteins, which exhibit this type of gate. It furthermore changed a component of fast gating at positive voltages, which was sensitive to mutations at this gate. A plausible explanation for this effect is that aPLs alter the orientation of the transmembrane helices in the bilayer. This may either prevent or destabilize the formation of intrahelical hydrogen bonds or corrupt orientation and hence the blocking effect of the aromatic side chains in the conductive pathway. This explanation is in good agreement with our finding that the effect of aPLs on gating in Kcv_S_ has some leaflet specificity. In asymmetrical bilayers, the aPL mostly increases the open probability when it is in the membrane leaflet that faces the cytosolic side. This corresponds to the side of the membrane where anionic lipids are present in native membranes (Fadeel and Xue, 2009; Rivel et al., 2019) and to the side of the channel where the critical gate is located (Fig. 1).

In this study, the viral channels serve as model systems that translate differences in the bilayer composition into functional readouts. At this point, it is not yet clear how insights from this orthogonal system can be extrapolated to physiologically relevant channels. However, it is important to note that the sensitivity of both conductance and open probability in the reference channel Kcv_S_ is half-maximal at a PS concentration of 15–25%. This is well in the range of PS concentrations (∼30%) that are reported for the inner leaflet of the plasma membrane of cells (Rivel et al., 2019). Hence, any dynamic change in the level of anionic lipids, which can occur in cells, for example, during tumorigenesis (Rivel et al., 2019) or as a result of controlled depletion of PS from the inner leaflet in apoptotic cell engulfment and myogenesis (Segawa et al., 2014; van den Eijnde et al., 2001), could in this way affect channel function.
